# Mesenchymal stem cell-derived interleukin-28 drives the selection of apoptosis resistant bone metastatic prostate cancer

**DOI:** 10.1038/s41467-021-20962-6

**Published:** 2021-02-01

**Authors:** Jeremy J. McGuire, Jeremy S. Frieling, Chen Hao Lo, Tao Li, Ayaz Muhammad, Harshani R. Lawrence, Nicholas J. Lawrence, Leah M. Cook, Conor C. Lynch

**Affiliations:** 1 Cancer Biology Ph.D. Program, University of South Florida, Tampa, FL USA; 2 Tumor Biology Department, H. Lee Moffitt Cancer Center and Research Institute, Tampa, FL USA; 3 Department of Drug Discovery, H. Lee Moffitt Cancer Center and Research Institute, Tampa, FL USA; 4 Department of Pathology and Microbiology, University of Nebraska Medical Center, Omaha, NE USA

**Keywords:** Bone cancer, Cancer microenvironment, Metastasis, Urological cancer

## Abstract

Bone metastatic prostate cancer (PCa) promotes mesenchymal stem cell (MSC) recruitment and their differentiation into osteoblasts. However, the effects of bone-marrow derived MSCs on PCa cells are less explored. Here, we report MSC-derived interleukin-28 (IL-28) triggers prostate cancer cell apoptosis via IL-28 receptor alpha (IL-28Rα)-STAT1 signaling. However, chronic exposure to MSCs drives the selection of prostate cancer cells that are resistant to IL-28-induced apoptosis and therapeutics such as docetaxel. Further, MSC-selected/IL-28-resistant prostate cancer cells grow at accelerated rates in bone. Acquired resistance to apoptosis is PCa cell intrinsic, and is associated with a shift in IL-28Rα signaling via STAT1 to STAT3. Notably, STAT3 ablation or inhibition impairs MSC-selected prostate cancer cell growth and survival. Thus, bone marrow MSCs drive the emergence of therapy-resistant bone metastatic prostate cancer yet this can be disabled by targeting STAT3.

## Abstract (web summary)

The effects of bone-marrow derived MSCs on prostate cancer cells remain unknown. Here the authors show that MSC-derived IL-28 induces prostate cancer cell apoptosis via IL-28Rα-STAT1 signalling, while acquired resistance to apoptosis is associated with a shift in IL-28Rα signalling via STAT1 to STAT3.

## Introduction

Recurrent metastatic prostate cancer (PCa) typically manifests in the skeleton where it can rapidly become resistant to standard treatments, including androgen deprivation therapies (ADTs) and chemotherapies^1–3^. Further, metastatic PCa disrupts bone homeostasis, provoking both bone destruction and abnormal bone formation, which significantly compromises the patient’s quality of life. We and others have reasoned that defining how prostate cancer cells establish and grow in the bone microenvironment may reveal new therapeutic targets for treatment^3–5^. This process is complex, where extravasation of PCa cells into bone results in their exposure to several cell types, including mesenchymal stem cells (MSCs)^6^, and metastatic cancer cells are known to localize in MSC-rich vascular and osteogenic niches^7,8^. Notably, PCa cells can promote MSC differentiation into bone forming osteoblasts^9^, yet little is known as to how MSCs may affect the maintenance, progression and resistance of bone metastatic prostate cancer.

MSCs can be both pro- and anti-tumorigenic depending on the stage of cancer progression and tissue context^10,11^. For example, MSCs have been shown to trigger multiple myeloma cell apoptosis via secretion of Fas Ligand (FasL)^12^. However, most studies have shown that MSCs contribute to cancer progression. In particular, MSCs recruited to the breast cancer microenvironment secrete chemokine ligand 5 (CCL5) that promotes cancer cell invasion and metastasis^13^. Further, in colorectal cancer MSC-derived neuroregulin 1 (NRG1) activates HER2/3 and AKT signaling to promote cancer cell survival, and MSC-derived IL-6 promotes the progression of hepatocellular carcinoma via activation of STAT3^14,15^. The effects MSCs have on tumorigenesis can also be dynamic, where for example, they impair progression during the initial stages of hepatocarcinoma but support later stages of the disease^16^. The opposing effects of MSCs on tumor progression may in part be explained by the tissue source. Specifically, MSCs can be derived from several sources—bone marrow, umbilical cord blood, peripheral blood, dental pulp, and adipose tissue—and MSCs from each tissue source differ in their capacity to either promote or inhibit cancer growth^17^.

Here, we report that bone-marrow derived MSCs produce IL-28 and that this provokes rapid apoptosis of bone metastatic prostate cancer cells via IL-28Rα-STAT1 signaling, which has known roles in apoptosis, and in anti-viral and immune responses^18^. However, we found that chronic exposure to MSCs leads to the selection of PCa populations that display a shift to IL-28Rα-STAT3 signaling and that are resistant to IL-28 induced apoptosis, and to conventional chemotherapies such as etoposide and docetaxel. Notably, STAT3 is generally considered to be pro-tumorigenic^19,20^ and is hyperactivated in bone metastatic prostate cancer^21,22^. In accord with these findings, treatment of MSC-selected PCa cells with a selective small molecule inhibitor of STAT3, S3I-201, impaired their growth and survival ex vivo and in vivo. Thus, the IL-28Rα-STAT3 signaling circuit represents an attractive and therapeutically tractable vulnerability for bone metastatic prostate cancer.

## Results

### MSCs have dynamic effects on prostate cancer cell growth

**Fig. 1:**
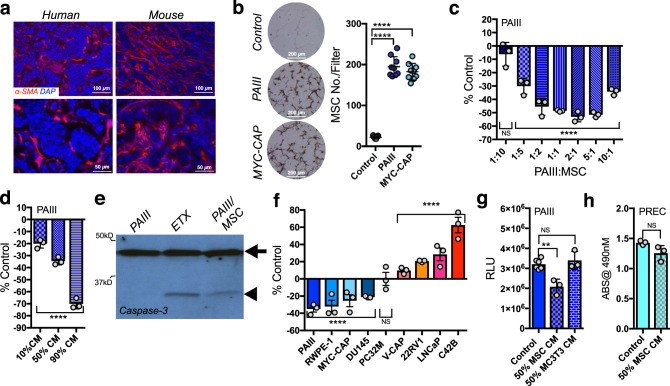
Bone marrow-derived MSC effects on prostate cancer cells. **a** Representative images (*n* ≥ 3 biologically independent samples) of α-Smooth muscle actin (SMA) staining of human and rodent bone metastatic prostate cancer. **b** MSC migration to prostate cancer cell conditioned media. Number of hematoxylin and eosin stained MSCs per filter (MC No./Filter; *n* = 3 fields of view from *n* = 3 biologically independent samples) were counted after 6 h of incubation. Representative photomicrographs of fields of view are shown. **c** Direct co-culture of MSCs and PAIII prostate cancer cells at various ratios of PAIII:MSC. Values calculated as percentage of respective PAIII controls seeded at the same density (% Control). Growth was determined by luminescence assay and relative light unit (RLU) measurement. **d** MSC conditioned media (CM) treatment of PAIII at varying ratios. Final concentration of serum was 10% for each condition. **e** Cleaved caspase-3 (arrow head) in PAIII cells treated for 6 h with MSC CM (50% ratio). Arrow indicates full-length caspase-3. Etoposide (ETX; 50 μM) was used as a positive control. These experiments were repeated twice with similar results. **f** MSC CM (50%) effects on prostate cancer cell line growth, relative to untreated controls. Growth measurements were performed by MTT or luminescence assay. **g** PAIII treated with MSC or osteoblast (MC3T3) CM. **h** Prostate epithelial cells (PREC) treated with MSC CM. MTT absorbance (ABS) was used as a readout for cell growth. Statistical analyses used include unpaired *t*-test (**c**, **d** and **f**–**h**) and ANOVA with multiple comparisons at 95% CI (**b**–**d** and **f**, **g**) with error bars representing the mean ± SD. All experiments were independently repeated (*n* = 3). Asterisks denotes statistical significance (*p ≤ 0.05, **p ≤ 0.01, ***p ≤ 0.001, ****p ≤ 0.0001) while NS denotes not significant.

Human and mouse bone metastatic prostate cancer specimens were initially assessed for MSC content by immunohistochemistry (IHC). As predicted^23–25^, there were abundant numbers of α-SMA positive MSCs in both human and mouse bone metastatic PCa (Fig. 1a). These findings were confirmed using additional markers for human (CD90) and mouse (nestin) MSCs (Supplementary Fig. 1)^25–27^. To assess the possible effects of MSCs on prostate cancer cells, we isolated mouse bone MSCs (CD29^+^SCA1^+^CD45^Neg^) and confirmed their stemness properties by performing osteoblastic, adipogenic, and chondrogenic differentiation assays (Supplementary Fig. 2). Using a modified Boyden chamber assay, we observed that MSCs are highly chemotactic to prostate cancer cell line-derived conditioned media compared to serum free control (Fig. 1b). To assess the potential effects of MSCs on prostate cancer cell fate, the rodent prostate cancer cell line PAIII was directly co-cultured with mouse MSCs^28,29^. Notably, MSCs significantly inhibited prostate cancer cell growth even down to 1:10 ratios of MSC to PAIII cells (Fig. 1c). Further, a dose-dependent inhibition of prostate cancer cell growth was evident when they were cultured with MSC conditioned media (CM); thus, a soluble MSC-derived factor suppresses prostate cancer cell growth (Fig. 1d). A significant rounding of PCa cells combined with detachment was noted within 6 h of culture with CM, suggesting that the MSCs triggered PCa cell apoptosis. Consistent with this, high levels of cleaved caspase-3 are manifest in MSC CM-treated PCa cells compared to controls within 6 h (Fig. 1e). This effect was not limited to PAIII PCa cells or to mouse MSC, as both mouse and human MSC conditioned medium impaired the growth of mouse Myc-CAP and human RWPE and DU145 prostate cancer cells (Fig. 1f). By contrast, culture in MSC CM increased the growth rates of V-CAP, 22RV1, LNCaP, and the LNCaP derived C4-2B prostate cancer cell lines compared to controls, underscoring the complexity of MSC effects on prostate cancer cell behavior (Fig. 1f). Of note, the negative effects on prostate cancer cell growth were MSC specific, as conditioned medium derived from osteoblasts failed to affect PCa cell growth (Fig. 1g). Finally, MSC CM also did not affect the growth of normal prostate epithelial cells (PREC; Fig. 1h).

### MSCs suppress the growth of bone metastatic prostate cancer in vivo

**Fig. 2:**
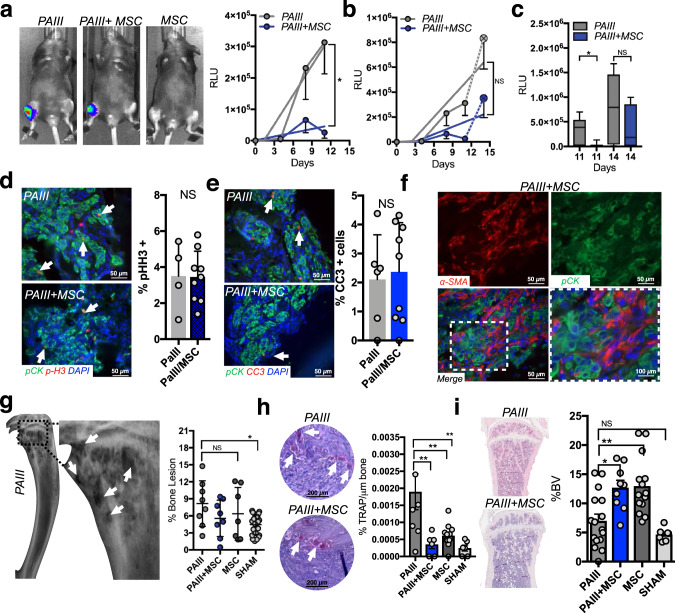
MSCs initially suppress prostate cancer growth in the bone microenvironment. **a**, **b** Prostate cancer growth (PAIII) overtime in the presence (1:1 ratio) or absence of MSCs (*n* = 8/group). An MSC-alone group was also included as a control (*n* = 7). Representative images of bioluminescence for each group at day 11 time point are shown. Graphs illustrate collected RLUs over time for each group thru days 11 and 14. Error bars represent mean ± SEM. Linear regression was used to determine significance at day 11 (*p* = 0.0324). **c** Analysis of RLU values at day 11 and 14 in the PAIII vs. PAIII + MSC group. Error bars represent the mean  ± SEM. **d**, **e** Ex vivo analyses from study endpoint of proliferative and apoptotic indices using phospho-histone H3 (p-H3; red arrows; **c**) and cleaved caspase 3 (CC3; red, arrows, **d**), respectively. Pan-cytokeratin (green) was used to identify prostate cancer cells. **f** Representative images (*n* ≥ 3 biologically independent samples) of smooth muscle actin staining (α-SMA; red) in tissues derived from the PAIII + MSC group. Pan-cytokeratin (pCK; green) was used to localize prostate cancer cells. Dashed box in merge represents area of magnification. **g** X-ray analysis of cancer-induced bone destruction. Representative X-ray from PAIII group is shown with dashed box defining area of magnification. Arrows indicate areas of cancer-induced bone destruction. The area of bone destruction was calculated as a percentage of total volume. **h** The number of osteoclasts (TRAcP positive; red, multinucleated; arrows) per μm of bone was calculated in non-sequential sections derived from the PAIII and PAIII + MSC groups. **i** Trabecular bone volume (BV) was measured via histomorphometry on non-sequential H&E multiple sections derived from each group and calculated as a percentage of total volume. Representative gross H&E images are illustrated from the PAIII and PAIII + MSC group. Representative micrographs (**d**, **e**, **g**, **i**) are derived from independent bones (≥ 3) from each study group. Statistical analyses were generated from one-way ANOVA with multiple comparisons at 95% CI. Asterisks denote statistical significance (*p ≤ 0.05, **p ≤ 0.01) while NS denotes not significant.

To examine the effects of MSCs on prostate cancer progression in bone, immunocompromised animals were intratibially injected with luciferase-expressing PAIII PCa cell (2 × 10^4^; *n* = 8) in the presence or absence of 1:1 ratio of mouse MSCs (2 × 10^4^; *n* = 8) to reflect our in vitro observations. A separate cohort of mice received MSCs alone (2 × 10^4^; *n* = 7). Contralateral limbs in each animal received sham injections that served as an internal baseline control. We have previously shown that the PAIII PCa model generates rapid mixed osteolytic/osteogenic responses over the course of ~15 days prior to breaching the cortical bone^28,30,31^. Using bioluminescence as a correlate of tumor growth over time, we noted that, similar to effects observed in vitro, PAIIII PCa growth was significantly suppressed by MSCs versus PAIII-alone cohort up to day 11 post-transplant (Fig. 2a). However, between day 11 and 14 we observed that the growth of the PAIII cancer cells co-injected with the MSCs rapidly accelerated (a 1260% increase in RLU from day 11–14) compared to the PAIII alone cohort (166% increase in RLU over the same time period) rendering the differences in tumor burden insignificant by day 14 (Fig. 2b, c). Analysis of proliferative (pHistone-H3) and apoptotic (cleaved caspase-3) indices confirmed increased proliferation and decreased apoptosis in the PAIII + MSC cohort with no statistical differences noted at the time of clinical endpoint compared to the PAIII-alone cohort (Fig. 2d, e). Analysis of MSC markers also demonstrated the persistence of the MSCs over time in the PAIII + MSC cohort (Fig. 2f).

X-ray analyses of cancer-associated bone disease revealed, as expected, significant areas of tumor-induced osteolysis in PAIII bearing tibia compared to sham controls^28,30^. However, quite strikingly, osteolysis was not evident in the PAIII + MSC cohort (Fig. 2g). Consistent with this observation, there were significantly higher numbers of TRAcP-positive mature multinucleated osteoclasts at the tumor-bone interface in the PAIII cohort compared to the PAIII + MSC, MSC or sham cohorts (Fig. 2h). Conversely, histomorphometry analysis demonstrated significantly higher levels of bone volume in the PAIII + MSC group compared to the PAIII cohort, which likely reflects increased MSC differentiation into osteoblasts (Fig. 2i).

### Chronic MSC exposure selects for apoptosis resistant prostate cancer

**Fig. 3:**
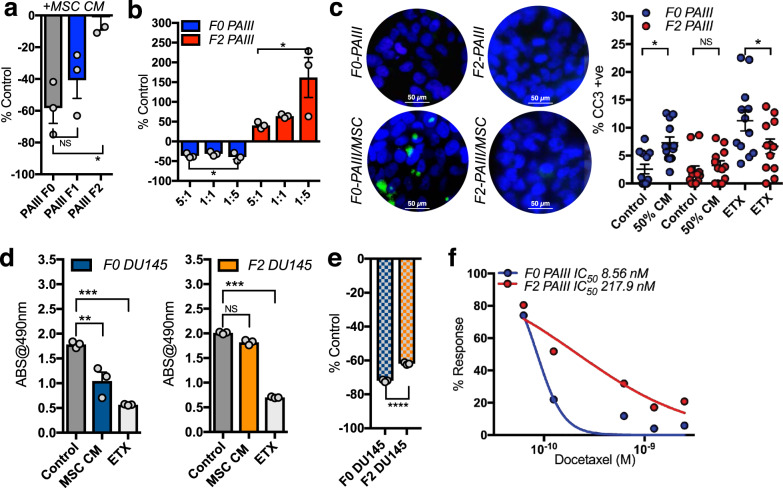
MSCs select for apoptotic resistant prostate cancer cell populations. **a** Growth of parental PAIIIs (F0) and MSC PAIII cell lines selected after one (F1) or two rounds (F2) of exposure to MSC conditioned media (50% concentration). Cell growth was calculated as a percentage of controls grown in the absence of MSC CM (*n* ≥ 3 biologically independent samples). **b** Direct co-culture of F0 and F2 PAIIIs with MSCs at varying ratios (MSC:PAIII). Data obtained from *n* ≥ 3 biologically independent samples. Cell growth was calculated as a percentage of F0 and F2 PAIIIs seeded at equivalent numbers in the absence of MSCs. **c** Immunofluorescence (IF) analysis of cleaved caspase-3 positivity (green) in F0 and F2 PAIII cell lines treated for 6 h with MSC CM. Graphs illustrate the number of cleaved caspase-3 positive cells as a ratio of total cell number (nuclear DAPI-blue). Data obtained from *n* ≥ 3 fields of view from at least three independent experiments. Etoposide (ETX; 50 μM) was used as a positive control. Data shown as mean ± SD. **d**, **e** MSC CM (50%) selection of apoptosis resistant DU-145 prostate cancer cells (F2) and the response to etoposide (ETX; 50 μM). Cell growth was calculated by MTT assay with absorbance (ABS) at 490 nm used as a correlate for cell number (*n* ≥ 3 biologically independent samples). **f** IC_50_ curves of PAIII F0 and MSC selected F2 cells treated with docetaxel for 48 h at a concentration range of 0–6.25 nM (*n* ≥ 3 biologically independent samples at each concentration used. Dots represent the mean combined with a nonlinear fit solid line. Error bars shown as mean ± SEM (**a**, **b**, **d**) or ±SD (**c**, **e**). Statistical analyses were generated from one-way ANOVA with multiple comparisons at 95% CI or unpaired *t*-test (**e**). Asterisks denotes statistical significance (*p ≤ 0.05, **p ≤ 0.01, ***p ≤ 0.001, ****p ≤ 0.0001) while NS denotes not significant.

In vitro, we observed some sensitive PCa cells treated with MSC CM persisted even after 24 h of exposure to CM and were able to form colonies. To assess if MSCs promote the selection of apoptosis resistant subpopulations, we exposed parental PAIII PCa cells (F0) to MSC CM for 72 h and allowed the surviving clones to grow out (F1). These cells underwent a consecutive round of MSC CM selection to yield a second MSC CM-educated population (F2). Cell growth analysis showed step-wise progressive enrichment of cancer cells resistant to growth inhibition by MSCs, F0 < F1 < F2 (Fig. 3a). Further, in direct co-culture experiments, with MSCs and cancer cells seeded at varying ratios, we observed an inhibitory effect on F0 parental cells but a proliferative effect on F2 MSC-selected cell lines (Fig. 3b). Consistent with this phenotype, immunofluorescence assays for cleaved caspase-3 demonstrated that apoptotic indices of F2 exposed to MSC CM were significantly lower than that of F0 cells (Fig. 3c). This effect was not limited to the PAIII PCa cells as chronic exposure to MSCs also selects for apoptosis resistant DU145 cells (Fig. 3d). We noted that both the F2 PAIII and F2 DU145 were also significantly more resistant to etoposide (ETX) induced apoptosis (Fig. 3c, e). To test if apoptosis resistance was solely due to MSC-derived factors or was more generalizable, we also examined the sensitivity of F0 and F2 cells to the chemotherapeutic drug docetaxel. Notably, MTT assays established that the IC_50_ docetaxel for F2 generated PAIII cells was 24-fold higher than that of the parental F0 cells (Fig. 3f). We further examined differences in the RNA profiles between the F0 and F2 PAIII and DU145 populations using RNA QuantSeq (Supplementary Fig. 3). Pathway and network analyses revealed that apoptotic and survival pathways were most impacted in the F2 prostate cancer cell populations underscoring that MSCs can drive the selection for apoptosis-resistant sub-populations of prostate cancer.

### MSCs accelerate prostate cancer progression in bone

**Fig. 4:**
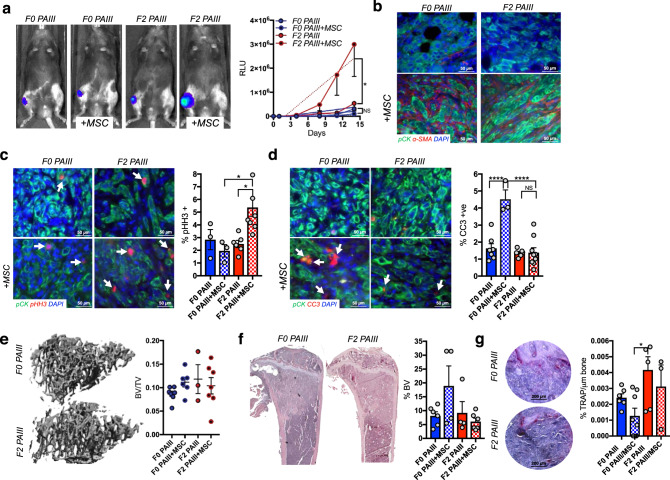
MSC selected prostate cancer cell growth is promoted rather than suppressed by the presence of MSCs. **a** Parental (F0 PAIII) and MSC selected (F2 PAIII) growth over time in the presence (1:1 ratio) or absence of MSCs (*n* ≥ 6/group). Representative images of bioluminescence in each group are shown at day 11 time point. Graphs illustrate collected RLUs over time for each group. Non-linear regression was used for statistical analysis with mean  ± SEM, *p* = 0.0052. **b** Representative images (n≥3 biologically independent samples) of smooth muscle actin staining (α-SMA; red) in tissues derived from the F0 and F2 groups in the presence or absence of MSCs. Pan-cytokeratin (pCK; green) was used to localize prostate cancer cells. Dashed box in merge represents area of magnification. **c**, **d** Ex vivo analyses from study endpoint of proliferative and apoptotic indices using phospohistone H3 (pHH3; red arrows; **b**) and cleaved caspase 3 (CC3; red, arrows, **b**), respectively. Pan-cytokeratin (green) was used to identify prostate cancer cells (*n* ≥ 3 fields of view from at least three independent experiments). **e** μCT scan analysis of cancer-induced bone destruction. Representative μCT images of the trabecular bone are shown for the F0 and F2 PAIII group. The trabecular bone volume was calculated as a ratio to total volume analyzed (BV/TV; *n* ≥ 3 bones from each group). **f** Trabecular bone volume (BV) was measured via histomorphometry on non-sequential H&E multiple sections derived from each group and calculated as a percentage of total volume (*n* ≥ 4 bones derived from each group). Representative gross H&E images are illustrated from the F0 and F2 groups. **g** The number of osteoclasts (TRAcP positive; red, multi-nucleated; arrows) per μm of bone was calculated in non-sequential sections derived from each group (*n* ≥ 3 fields of view from at least three independent experiments). For all graphs shown, error bars represent mean ± SEM, statistical analyses were performed by one-way ANOVA with multiple comparisons. Asterisks denotes statistical significance (*p ≤ 0.05, ****p ≤ 0.0001) while NS denotes not significant.

To assess the effects of MSC selection on bone metastatic prostate cancer disease progression, immunocompromised animals were intratibially injected with luciferase-expressing F0 and F2 PAIII cell lines (2 × 10^4^; *n* ≥ 7) in the presence or absence of mouse MSCs (2 × 10^4^; *n* ≥ 7). Sham injected contralateral limbs in each animal served as an internal baseline control. Analysis of bioluminescence over time showed that that the F2 cell line, rather than being suppressed by MSCs, grew at significantly faster rates compared to all other cohorts (Fig. 4a). Interestingly, F0 and F2 PAIII-derived tumors grew at comparable rates, suggesting that MSCs drive the accelerated growth effects of the F2 PAIII cell line in vivo. In accord with the phenotypes manifest in vivo, IHC analysis of α-SMA demonstrated the persistence of the MSCs in the cancer-bone microenvironment over the course of the studies (Fig. 4b). Ex vivo analyses of the proliferative indices of F0 versus F2 PAIII cells agreed with the in vivo bioluminescence readouts, where there were significantly higher rates of proliferation in the F2 PAIII cells when grown with MSC compared to all other groups (Fig. 4c). Further, analysis of apoptotic indices showed significantly more apoptosis occurring in the F0 MSC treated group (Fig. 4d). Although there was little impact of MSC on parental versus F2 PAIII prostate cancer cells on associated bone disease, as measured by μCT or histomorphometry analyses (Fig. 4e, f), there were significantly higher numbers of osteoclasts in the F2 PAIII-alone cohort (Fig. 4g). While this is indicative of higher rates of bone remodeling, it did not manifest as increased cancer-induced bone destruction.

**Fig. 5:**
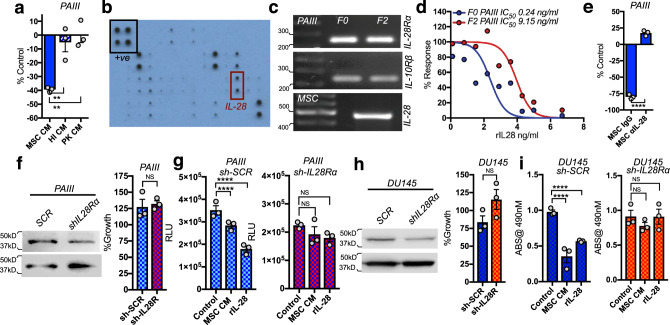
MSC-derived IL-28 directs PCa apoptosis. **a** PAIII growth (F0) in response to treatment with MSC CM, heat-inactivated (HI) MSC CM, or proteinase-K (PK) treated MSC CM. **b** Cytokine Array of MSC CM. Black box indicates positive control (+ve), red box indicates IL-28. **c** RT-PCR analysis of PAIII (F0 and F2) of IL28Rα, IL-10Rβ and IL-28 expression. Molecular weights in base pairs are shown. **d** Growth of PAIII (F0) in MSC CM immune-depleted of IL-28 (MSC αIL-28). IgG was used as negative control (MSC IgG). Growth is expressed as a percentage of non-treated cells. **e** Treatment of PAIII F0 and F2 cell lines with the indicated concentrations of recombinant IL-28 (rIL-28) for 48 h. **f** Growth of IL-28Rα silenced (sh-IL28R) and scrambled control (sh-SCR) compared to parental PAIII cell lines. **g**, **h** Control (sh-SCR) and IL-28Rα (sh-IL28R) PAIII and DU145 growth in MSC CM or rIL-28 as measured by luminescence assay and relative light unit (RLU) measurement or MTT assay. Experiments were repeated on at least two (**c**, **f**, **h**) or three (**a**, **e**–**i**) occasions. Error bars represent the mean ± SEM. Statistical analyses were performed by one-way ANOVA with multiple comparisons at 95% CI (**a**, **g**, **i**) or unpaired *t*-test (**e**, **f**, **h**). Asterisks denotes statistical significance (**p ≤ 0.01, ****p ≤ 0.0001) while NS denotes not significant.

Proteinase-K treatment and heat inactivation of the MSC CM revealed that a soluble protein was necessary for the observed apoptotic effects in co-culture with prostate cancer cells (Fig. 5a). To identify potential apoptosis inducing factors, cytokine arrays were performed, which revealed that Fas ligand (FasL), galectin 1 (GAL1), and interleukin-28 (IL-28) were specifically found in MSC-derived conditioned media (Fig. 5b). IL-28 is known to trigger apoptosis but has not been explored in the context of bone metastatic prostate cancer^32–35^. Analysis of publicly available datasets revealed that IL-28, and its cognate receptors IL28Rα and IL10Rβ were expressed in prostate carcinoma compared to prostate glandular epithelium (Supplementary Fig. 4). We also demonstrated the presence of the IL-28Rα in pan-cytokeratin positive prostate cancer cells in human samples of bone metastatic prostate cancer (Supplementary Figure 4). Next, we tested if PCa cells were sensitive to IL-28 induced apoptosis. Both F0 and F2 PAIII prostate cancer cells expressed IL-28Rα and IL-10Rβ, whereas MSCs expressed IL-28 (Fig. 5c). Notably, using recombinant IL-28, F2 PAIII cells were significantly more resistant to IL-28 mediated cell death, with an IC_50_ > 35-fold higher than that observed in the parental F0 cell lines (F0 IC_50_ = 244 pg/ml, F2 IC_50_ = 9145 pg/ml; Fig. 5d). Further, addition of IL-28 neutralizing antibody to MSC CM, but not of isotype-matched IgG, ablated MSC-induced apoptosis of parental F0 PAIII cells (Fig. 5e). Similarly, efficient shRNA-directed knockdown of IL-28Rα expression in parental PAIII cells blocked MSC- and IL-28-induced apoptosis without affecting the growth of these cells (Fig. 5f, g). In contrast, MSC CM or recombinant IL-28 triggered rapid decreases in cell number in scrambled shRNA control cells (Fig. 5g). Similar findings were observed following shRNA-directed knockdown of IL-28Rα in DU145 PCa cells (Fig. 5h, i), confirming the role of MSC-derived IL-28 in mediating the observed apoptotic effect.

### Selection for MSC-derived resistance leads to IL-28-STAT3 signaling

**Fig. 6:**
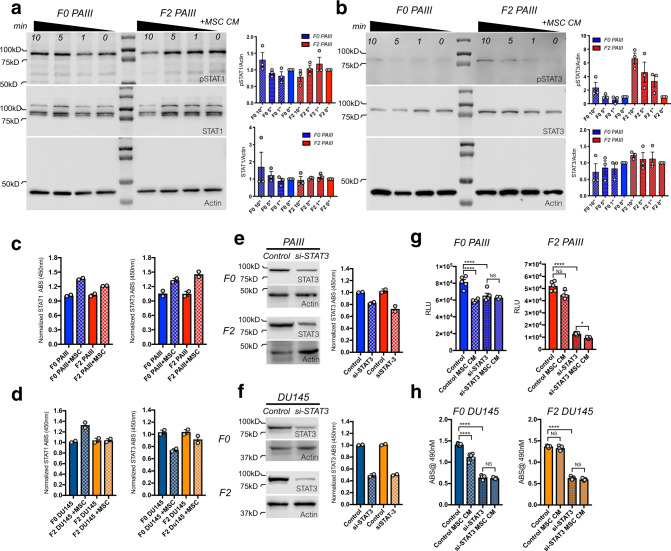
Elevated STAT3 signaling in MSC selected prostate cancer cell lines. **a**, **b** pSTAT1 (**a**) and pSTAT3 (**b**) levels at baseline and in response to MSC CM (50%) over a 10 min (min) period in PAIII parental (F0) and MSC selected (F2) cell lines. Molecular weights are shown in kDa. Actin was used as a loading control. For densitometry, pSTAT and STAT levels were each normalized to their respective actin controls and time responses for F0 and F2 are compared to their respective 0’ minute controls that were set at a normalized value of 1. All experiments were repeated on at least three separate occasions with similar results. **c**, **d** STAT1 and STAT3 DNA binding activity in the PAIII (**c**) and DU145 (**d**) F0 and F2 cell lines was measured in response to MSC CM for 30 min. Results obtained via absorbance (ABS@450 nm) were normalized to respective controls. **e**, **f** STAT3 was silenced (si-STAT3) in PAIII (**e**) and DU145 (**f**) F0 parental and F2 MSC-selected cell lines and the resultant impact on STAT3 activity was measured. Blots show total STAT3. Experiments were independently repeated with similar results. **g**, **h** The effect of STAT3 silencing on PAIII (**g**) and DU145 (**h**) cell growth in the presence or absence of MSC CM compared to control treated cells using luminescence assay and relative light unit (RLU) measurement or MTT assay (*n* ≥ 3 biologically independent samples). Molecular weights are shown in kDa. Error bars in graphs represent the mean ± SEM and statistical analyses were performed by one-way ANOVA with multiple comparisons at 95% CI. Asterisks denote statistical significance (*p ≤ 0.05, **p ≤ 0.01, ***p ≤ 0.001, ****p ≤ 0.0001) while NS denotes not significant.

Despite clear differences in sensitivity to IL-28-induced apoptosis, levels of IL-28Rα were similar in F0 versus F2 cell lines, nor did we observe any differences in receptor induction in response to MSC CM over time (Supplementary Fig. 5). IL-28Rα induces phosphorylation of STATs via JAK/TYK kinase activation^18^. In the context of cancer, STAT1 is considered a tumor suppressor while STAT3 is often associated with tumor progression^19^. Consistent with previous publications^21^, analysis of human bone metastatic specimens (*n* = 10) demonstrated phosphorylated STAT3 in pan cytokeratin positive prostate cancer cells (Supplementary Fig. 6). We therefore assessed the activity of STAT1 and STAT3 in our PCa cell models by monitoring their total protein levels and phosphorylation status in response to MSC CM over time. In independent experiments, we noted increases in total STAT1 and pSTAT1 in the F0 PAIII compared to F2 PAIII in response to MSC CM while conversely, we observed increased total STAT3 and pSTAT3 (Tyr 705) in the F2 PAIII cells compared F0 cells (Fig. 6a, b). These results were mirrored in DU145 cells (Supplementary Fig. 7). Using quantitative STAT activity assays, we also observed that MSC conditioned media enhanced STAT1 activity in the PAIII and DU145 F0 cell lines compared to F2 response (Fig. 6c, d). Conversely, we identified that MSC CM increased STAT3 activity in the PAIII F2 cell lines (Fig. 6c) but for DU145, MSC CM lowered STAT3 activity in the F0 cells while having no effect on the F2 population (Fig. 6d). These data suggest that the MSC selected apoptosis resistant prostate cancer cells favor STAT3 over STAT1 signaling. Finally, in accord with its known pro-tumorigenic roles^36^, siRNA-directed knockdown of STAT3 (Fig. 6e, g), reduced the growth of all PCa cells with the addition of MSC CM to the STAT3 silenced cells having little further effect on cell viability (Fig. 6f, h).

### STAT3 inhibition mitigates the growth of MSC-educated prostate cancer cells

**Fig. 7:**
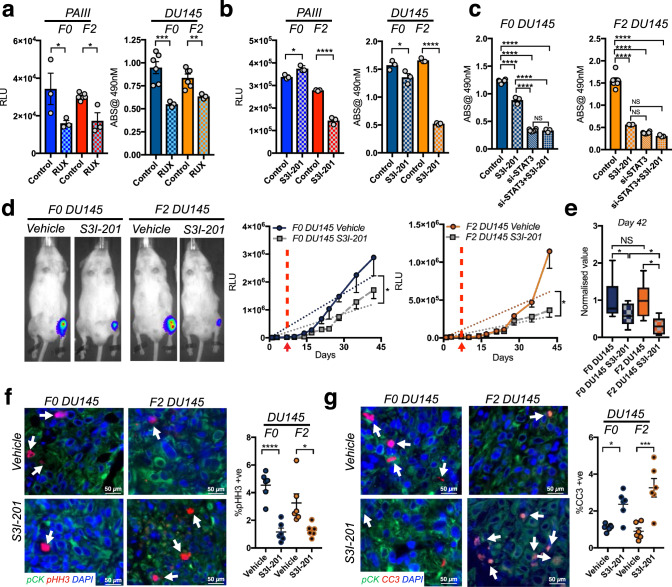
STAT3 inhibition impairs the growth of MSC-selected prostate cancer in vitro and in vivo. **a**, **b** Parental (F0) and MSC-selected (F2) cell lines treated with vehicle control (Control) or the JAK2 inhibitor ruxolitinib (RUX)/STAT3 inhibitor (S3I-201) for 24 h. **c** F0 and F2 DU145 control (scr-siRNA) or STAT3 silenced (si-STAT3) cells treated with vehicle or S3I-201 for 24 h. **d** F0 and F2 DU145 growth over time in the presence or absence of STAT3 inhibitor, S3I-201 (*n* = 10/group). Representative images of bioluminescence in each group are shown at day 35-time point. Arrow and dashed line represent time of treatment initiation. Graphs illustrate collected RLUs over time for each group. Error bars represent the mean ± SEM and linear regression was used for statistical analysis. **e** S3I-201 effect on F0 and F2 DU145 at day 42 normalized to respective controls. Box and whisker plots show min to max values obtained. **f**, **g** Ex vivo analyses from study endpoint of proliferative and apoptotic indices using phospohistone H3 (pHH3; red arrows; **f**) and cleaved caspase 3 (CC3; red, arrows, **g**), respectively. Pan-cytokeratin (green) was used to identify prostate cancer cells. Error bars represent the mean  ± SEM. Experiments (**a**–**d**) were repeated on at least three separate occasions with similar results. Error bars in graphs represent the mean ± SEM. Statistical analyses were performed by one-way ANOVA with multiple comparisons at 95% CI. Asterisks denote statistical significance (*p ≤ 0.05, **p ≤ 0.01, ***p ≤ 0.001, ****p ≤ 0.0001) while NS denotes not significant.

JAKs mediate IL-28Rα and IL-10β signal transduction^33^, and several JAK inhibitors have entered the clinical setting^37^. Treatment with the JAK1/JAK2 inhibitor ruxolitinib reduced STAT3 phosphorylation and impaired the growth of both F0 and F2 MSC-selected F2 PAIII and DU145 cells (Fig. 7a and Supplementary Fig. 7). Of note, in this system, ruxolitinib and S3I-201 had little to no effect on STAT1 activity (Supplementary Fig. 8). These data suggest likely differing pathway activities for IL-28 in the context of prostate cancer cell signaling.

We next focused on specific inhibition of STAT3. Translational efforts in this area have proven challenging, but the development of STAT3 inhibitors is important given high levels of pSTAT3 in advanced cancers^19–21,36^. In collaborative efforts, we previously developed S3I-201, an inhibitor that prevents STAT3 dimerization, and demonstrated its efficacy in treating breast cancer growth in vivo with no noted toxicities^38^. Notably, treatment with S3I-201 selectively impaired the growth of the MSC-selected F2 PAIII and DU145 cell lines with little effects on F0 parental cell lines (Fig. 7b). The specificity of S3I-201 was confirmed in STAT3-silenced F2 cell lines, where treatment led to no further decrease in cell growth than that provoked by knockdown of STAT3 alone (Fig. 7c).

To test the sensitivity of the F2 generated prostate cancer cell lines to STAT3 inhibition, we assessed the efficacy of the S3I-201 inhibitor in vivo. Mice were intratibially injected with luciferase expressing F0 and F2 DU145 cell lines and randomized after 7 days into vehicle control (*n* = 10) or S3I-201 groups (*n* = 10). Over time we observed that S3I-201 significantly reduced the intraosseous growth of the F0 and F2 groups compared to their respective controls (Fig. 7d). Normalization to controls at study endpoint further demonstrated that S3I-201 was more effective in reducing the growth of the F2 DU145 compared to the F0 DU145 population (Fig. 7e). Ex vivo analyses of the injected tibia revealed that S3I-201 significantly inhibited the proliferation of the F0 and F2 PCa cells (pHistone H3) while augmenting the apoptosis index (cleaved caspase-3), particularly in the F2 group (Fig. 7f, g). Consistent with reduced cancer growth, S3I-201 also protected against cancer-associated bone disease as measured by μCT, X-ray, histomorphometry and TRAcP staining (Supplementary Fig. 9). S3I-201 also effectively limited the growth of F2 PAIII PCa cell lines in vivo and significantly mitigated proliferation (pHistone H3) (Supplementary Fig. 10). Despite decreased tumor growth, we detected no differences in cancer-associated bone disease but this may be due to the rapid nature of the PAIII model (2 weeks) compared to the DU145 model (6 weeks). Importantly, however, our findings establish that MSC-selected apoptosis resistant F2 prostate cancer cells are highly sensitive to STAT3 inhibition in vivo.

## Discussion

Bone metastatic prostate cancer remains incurable and affects the majority of men diagnosed with recurrent castrate resistant disease. These metastatic lesions are associated with extensive bone remodeling, which generates factors that promote the growth, survival and persistence of prostate cancer cells in the face of applied systemic ADT and chemotherapies^3,39,40^. Understanding how osteoblasts and osteoclasts mediate these effects has led to the development of therapies that target the microenvironment, such as the RANKL binding monoclonal antibody denosumab and nitrogen containing bisphosphonates that block osteoclast formation and activity^3^. While effective in preventing skeletal pathologies such as fracture, these therapies are, unfortunately, largely palliative.

The heavy infiltrates of osteoblast progenitor MSCs in bone metastatic prostate cancer we observed is not surprising considering the bony nature of these lesions, and that prostate cancers are known to drive MSCs into an osteoblastic phenotype^9,17,24,25,41^. However, the dynamic effects of MSCs in promoting apoptosis and hence the selection for therapy resistance disease via MSC-derived IL-28 was unexpected, where MSC-derived IL-28-induced apoptosis drives the selection for broadly resistant subpopulations that are growth stimulated rather than repressed by MSCs. Further, the switch from apoptosis sensitivity to resistance was accompanied by a shift in STAT1 to STAT3 signaling that we have shown represents a vulnerability. Our in vivo studies demonstrated that STAT3 inhibition with S3I-201, was effective in significantly reducing the growth of MSC-selected prostate cancer. Our working hypothesis is that upon entry into the bone marrow, metastatic prostate cancer cells interact with resident MSCs resulting in the majority of the cancer cells being eliminated. Over time, however, MSCs eventually select for prostate cancer cells that are refractory to IL-28 induced apoptosis and have increased STAT3 signaling. Interestingly, we found that the MSC selected prostate cancer cells are also cross resistant to chemotherapies such as etoposide and docetaxel suggesting that STAT3 inhibition could be a viable route to resensitizing bone metastatic prostate cancer that is refractory to chemotherapy.

The tumor-promoting properties of MSCs revealed herein are largely in accord with studies establishing that MSCs contribute to, and in some cases are necessary for, tumor progression. For example, in breast cancer, intratumoral MSCs express chemokine ligand 5 (CCL5) that promotes invasion and metastasis^13^, and co-inoculation with MSCs promotes progression and metastasis of both osteosarcoma and ovarian cancer^42,43^. Mechanisms ascribed to these effects include angiogenesis and protection from hypoxia via the secretion of vascular endothelial growth factor (VEGF), immune suppression/modulation, the suppression of apoptosis, and the induction of the epithelial to mesenchymal transition (EMT) program^44,45^. Consistent with the positive effect of MSCs on cancer cell growth, we have shown here that bone-marrow derived MSCs enhance the proliferation of a subset of metastatic prostate cancer cell lines, such as C4-2B, which of note is derived from in vivo LNCaP bone metastases and has endogenously elevated levels of STAT3 activity^46^. Conversely, MSCs can have tumor suppressive effects in hepatoma and glioma where they induce cell-cycle arrest and apoptosis^47^. MSC-derived interferons (IFNs) have been shown to trigger apoptosis MCF-7 breast cancer cells via activation of STAT1^48^. Notably, a similar mechanism appears manifest in prostate cancer cells that are vulnerable to MSC-derived IL-28 induced apoptosis via STAT1 activation.

As documented here, IL-28 joins a cast of other cytokines that activate STAT3, including IL-11, leukemia inhibitory factor (LIF), oncostatin M (OSM) and IL-31^49,50^. STAT3 has clear roles in driving cancer progression and therapy resistance but rather little has been described regarding the tumorigenic roles of IL-28^19,21,51–53^. In a mouse model of B16 melanoma progression, IL-28 reduces tumorigenicity but how this occurs is not clear and could represent effects on immune surveillance^54^. This remains an important area for future investigation in bone metastatic prostate cancer, where syngeneic cell lines in immunocompetent animals (TRAMP-C3, MyC-CAP, RM1) will allow for the study of MSCs and IL-28 on infiltrating immune cells.

In addition to IL-28 our cytokine array analysis also revealed the presence of additional apoptotic factors in the MSC CM, such as FasL. Indeed, release of FasL via matrix metalloproteinase-7 (MMP-7) has been shown to be responsible for prostate epithelial cell apoptosis during involution of the organ subsequent to castration^55^. This phenomenon has also been demonstrated in the mammary gland where expression of a MMP-7 transgene in mammary epithelium augments apoptosis and involution at weaning but eventually provokes the development of hyperplasia in multiparous mice^56^. Further, chronic exposure of breast cancer cells to soluble FasL selects for apoptosis resistant subpopulations^57^. However, in our models, though PAIII are sensitive to FasL-induced apoptosis (Supplementary Fig. S11), immunodepletion of IL-28 completely abrogates the apoptotic effects of MSC CM (Fig. 5e).

MSC-selected apoptosis-resistant prostate cancer cells have elevated pSTAT3 and hyperactivation of STAT3 signaling occurs in many human cancers where it connotes poor prognosis and resistance to chemotherapy and radiation therapy^52,58^. In bone metastatic prostate cancer, IHC analyses have identified that the majority of cases studied are positive for STAT3, and kinome profiling have shown elevated activity of JAK2, which phosphorylates STAT3 at Tyr705 resulting in head to tail dimerization, translocation to the nucleus and binding to the promoters of target survival genes such as BCL-xL and survivin^59^. Here we have shown preferential STAT3 activity in MSC/IL-28-resistant prostate cancer that confers resistance to chemotherapies used to treat bone metastatic prostate cancer. Since there is no change in the level of IL28Rα or IL10Rβ, it is not clear at this juncture what causes the termination of STAT1 phosphorylation in the MSC educated prostate cancer cells. STAT inactivation can be controlled by a number of mechanisms that might be altered between the F0 and F2 populations including protein tyrosine phosphatases that depending on kinetics and cellular compartmentalization may preferentially dephosphorylate specific STATs^60–62^. Suppressors of cytokine signaling (SOCS) and protein inhibitors of activated STATs (PIAS) can also act as inhibitors of STATs or direct them for protein degradation^60^. For example, STAT interacting LIM protein (SLIM) acts as a ubiquitin E3 ligase and can direct STATs, in particular STAT1, for proteasomal degradation^63^. Further, STAT3 itself can bind to STAT1 and prevent the transcription of STAT1 target gene suggesting that STAT3 activity in the F2 cells could further limit STAT1 activity^64^. RNA QuantSeq analysis comparing the MSC educated F2 cell lines to their parental counterparts also demonstrated that genes such as SPRY2 (a negative regulator of interferon signaling and IFN inducible genes) were significantly downregulated in MSC educated cells. Further, bioinformatic analyses of the most downregulated pathways and networks in the MSC educated PAIII and DU145 F2 populations are; apoptosis regulated by mitochondrial proteins and apoptotic mitochondria respectively (Supplementary Fig. 3). Interestingly, STAT3 has been shown to accumulate in the mitochondria^65^, prevent mitochondrial mediated apoptosis and is linked to enhanced survival and drug resistance^66,67^. We should also note that deletion of STAT3 and PTEN in genetically engineered mouse models promoted prostate cancer progression and soft tissue metastasis (liver and lungs)^53^. Further, STAT3 expression was detected in only ~40% of metastases but the location of the metastases was not obvious. The tumor microenvironmental context of the metastases can have profound effects on cancer cell behavior. Given that other reports have identified high STAT3 positivity in bone metastatic prostate cancer^21^ and that the majority of prostate cancer in humans metastasizes to the skeleton^3^, we believe that STAT3 may be an important regulator of prostate cancer progression, specifically in bone. Importantly, STAT3 is also revealed here to be a targetable vulnerability that disables the growth of bone metastatic PCa, suggesting that STAT3 inhibitors such S3I-201^38^, which appears well-tolerated in pre-clinical studies, and have potential in the prostate cancer clinic. Alternatively, FDA approved JAK2 inhibitors such as ruxolitinib could be considered for the treatment of metastatic CRPC patients, yet to date ruxolitinib has shown only very modest effects in phase II clinical trials (NCT00638378), suggesting more specific targeting of JAK2 or better delivery methods are required to realize the therapeutic potential of JAK2 inhibition.

In conclusion, we have shown that bone marrow-derived MSCs drive the emergence of apoptosis resistant subpopulations of prostate cancer cells via the chronic exposure to MSC-derived IL-28, and that this is associated with increases in STAT3 activity that are necessary for the maintenance of bone metastatic PCa. Our findings also indicate that the application of STAT3 inhibitors may resensitize prostate cancer cells to chemotherapy and that, given the role of STAT3 activity in the progression of a wide range of cancers, the mechanism described herein may have broad applicability to other skeletal malignancies and/or metastases.

## Methods

### Cell lines, culture, and animals

LNCaP (Cat # CRL-1740), DU145 (HTB-81), MC3T3 (CRL-2594), RWPE-1 (CRL-11609), V-CAP (CRL-2876), 22RV1 (CRL-2505) MyC-CaP (CRL-3255) cell lines were purchased from the ATCC. PC3-2M cells were purchased from Perkin Elmer, PrEC prostate epithelial cells (CC-2555) and human MSCs (PT-2501) were purchased from Lonza while PAIII cells [27] and C4-2B [67] were kindly donated. All cell lines were periodically mycoplasma tested (CUL001B, R&D Systems) and short tandem repeat (STR) verified at the Moffitt Clinical Translational Research Core. Cell lines were passaged in recommended culture medium supplemented with 10% fetal calf serum (FCS). Isolation and culture methods for mesenchymal stem cells (MSCs) were adapted from previously published protocols^68^. Briefly, hind limbs were collected from tumor-naive 4–6-week-old male C57/BL6 Rag2−/− mice in sterile PBS. Following removal of muscle tissue, epiphyses were removed and bone marrow flushed three times with sterile PBS to deplete the hematopoietic cells. Flushed bones were then cut into 1–3 mm chips, digested with 1 mg/mL collagenase II (Invitrogen) in α-MEM with 15% FBS, and shaken at 150 RPM for 1 h at 37 °C. Digested bone fragments were grown in 6-well tissue culture plates in α-MEM with 15% FCS. Medium was changed every 3 days. For direct co-culture experiments, cancer cells expressing luciferase were cultured with murine MSCs at multiple ratios seeded for a total density of 2×10^4^ in 48-well plates. Co-cultures were incubated for 48 h, and PAIII growth was measured by bioluminescence using the Promega Luciferase Assay System (E1500) per the manufacturer’s instructions. For analyses assessing the growth of cancer cell lines in response to MSC CM, MTT assays were used. Prostate cancer cell lines were plated in 96-well plates at a density of 1×10^4^ cells/well and treated with MSC conditioned media. Cell viability was measured at 48 h by the MTT assay following the manufacturer’s instructions (CellTiter 96, #G3582, Pierce) by measuring absorbance at 490 nm after 4 h of incubation at 37 °C.

### Migration assay

Cells were serum starved for 2 h before trypsinizing and seeding (2 × 10^5^ cells) into upper chambers of 24-well Transwell membrane assay system (Corning). Lower chambers were prepared either 650 µl of either serum free, 1% serum or MSC CM. All conditions were performed in triplicate and incubated for 5 h at 37 °C. After incubation, upper chambers were rinsed in DI water followed by 1× PBS and fixation in methanol at −20 °C for 20 min. The chambers were then rinsed in water followed by 1× PBS, and non-migrated cells removed by gentle scrubbing with cotton tip applicators. After rinsing in DI water, membranes were stained with hematoxylin and dehydrated with 100% ethanol. The membranes were air dried dry and excised using a scalpel before mounting on slides with Permount (Fisher Cat # SP15-100). Three fields of view from each membrane were acquired using brightfield microscopy and the number of migrated cells per field counted.

### Intratibial tumor studies

Mice were purchased from Jackson Laboratory. All animal experiments were performed with IACUC approval (R1762, CCL) and in accordance with the guidelines set forth in the Guidelines for the Care and Use of Laboratory Animals published by the National Institutes of Health. 6-week-old male Rag2−/− mice were intratibially injected with luciferase-expressing PAIII or murine MSCs (2 × 10^4^ in 20 μl of sterile saline), or PAIIIs and murine MSCs 1:1 for a total of (4 × 10^4^ in 20 µl of sterile saline). 6-week-old NSG mice were intratibially injected with luciferase expressing DU145 cells (5 × 10^5^ in 20 μl of sterile saline). Contralateral limbs were injected with saline to control for injury induced changes. For S3I-201 in vivo studies, tumors were allowed to establish for 3 days before randomizing groups for treatment with S3I-201 (5 mg/kg) or sterile PBS administered by intraperitoneal injections in 200 µl volumes every other day. Bioluminescence for all studies was measured twice weekly as a correlate of tumor growth (IVIS™ Perkin Elmer). Mice that showed tumor growth outside of the bone compartment were excluded from all analyses.

### Cell line conditioned media collection and treatment

MSCs were cultured in T-75 cell culture flasks until 80–90% confluent. Each flask was rinsed three times with sterile 1× PBS before adding 5 mL of serum free medium. After incubating for 24 h, conditioned media (CM) was centrifuged at 4000 × *g* for 5 min, transferred to a new tube, and stored at 4 °C. Fresh CM was collected weekly and stored at 4 °C. To test if the MSC-derived factor responsible for prostate cancer apoptosis was a soluble protein, MSC CM and matched control serum free αMEM were either heat inactivated at 95 °C for 30 min or treated with 100 µg/ml proteinase K followed by heat inactivation 95 °C for 30 min. PAIII cells were seeded at 3 × 10^4^ and treated with each respective media type for 24 h. Cell growth was analyzed by luminescence assay (Promega Luciferase Assay System, Cat # E1500).

For IL-28 neutralization studies; PAIII cells were seeded in white wall, solid bottom 96-well plates (5 × 10^4^ cells/well) and incubated for 24 h before treatment with either MSC CM or serum free media control containing either 10 µg/ml of neutralizing antibody (R&D, AF1789) or normal goat IGG (R&D, AB108C). Cells were incubated for 48 h, and growth was analyzed using the Promega Luciferase Assay System, Cat# E1500 following the manufacturer’s instructions.

For docetaxel, recombinant IL-28, JAK2 inhibition (ruxolitinib) and STAT3 inhibition (S3I-201) parental (F0) or MSC-selected prostate cancer cells (F2) cells were seeded (5×10^3^) in 96 well plates and treated as follows. Docetaxel; 0, 0.0625, 0.125, 0.625, 1.25, 2.5, and 6.25 nM for 48 h. Recombinant IL-28 (R&D mIL-28B, Catalog #1789-ML); 0, 0.5, 1, 2, 4, 8, 16, 32, 64, and 128 ng/ml. Ruxolitinb; 0, 0.0001, 0.001, 0.01, 0.1, 1, and 10 μM. S3I-201 0, 0.1, 0.5, 1, 5, 10, 25, 50, 100, and 150 μM. Cell growth was assayed via luminescence or MTT assay.

### Ex vivo bone analysis

Tibias were collected and fixed in 10% formalin for 24–48 h and then transferred to 50% ethanol. Radiographic images (Faxitron, X-ray Corp) were obtained using energy of 35kVp and an exposure time of 8 milliseconds. The spatial resolution is 10 lp/mm (48 μm). The tumor volume (TuV) was calculated as a function of the total tissue volume (TV) of the tibial medullary canal using ImageJ software. For μCT analysis, the proximal tibia metaphyses were scanned (μCT-40; Scanco Medical). An evaluation of trabecular bone structural parameters was performed in a region that consisted of 1 mm starting at 500 μm from the growth plate. A three-dimensional cubical voxel model of bone was built and calculations were made for relative bone volume per total volume and trabecular number. After X-ray and μCT analysis, tibias were decalcified (14% EDTA, pH 7.4, 3 weeks), processed, and paraffin embedded.

### Immunofluorescence

For paraffin embedded tissues, slides were dewaxed and rehydrated to water. Antigen retrieval was performed by heat (1× Tris EDTA pH 8.0 in pressure cooker, 5 min). Proteinase K antigen retrieval was used for αSMA staining (7.5 min at room temperature 5 mL 2 M Tris pH 7.5, 5 mL 0.2 M EDTA, 190 mL ddH2O and 400 µL of 10 mg/mL proteinase K). Slides were blocked in 10% goat serum in 1× TBS for 1 h room temperature. Primary antibodies (Pan Cytokeratin, Sigma-Aldrich Cat # C2562. 1:200 dilution; Phospho-Histone H3, Cell Signaling Cat #9701L, 1:200 dilution; Cleaved Caspase 3, Cell Signaling Cat #9661S, 1:200 dilution; Alpha Smooth Muscle Actin (1A4), Cell Signaling Cat # 48938, 1:200 dilution IL-28Rα, Bioss, Cat # ABIN1387718) Cell Signaling Cat#9145 pSTAT3 Tyr705 (D3A7) 1:100 dilution, abcam Cat #ab92574 CD90/Thy1 [EPR3132] 1:100 and R&D Cat# MAB2636 Mouse/Rat Nestin 1:50 were diluted in 10% normal goat serum (Vector Laboratories Cat # S-1000) and incubated overnight at 4 °C in a humidified chamber. After 3 washes in 1× TBST followed by 1 wash in 1× TBS, secondary antibodies (Alexa FluorTM Goat Anti Rabbit 568, (Thermo Fisher Scientific #A-11011); Alexa Fluor Goat Anti Mouse 488, (Thermo Fisher Scientific #A32723) were incubated at a 1:1000 dilution in 10% normal goat serum for 1 h at room temperature. Slides were washed three times in 1× TBS and mounted using Vectashield Antifade Mounting Medium with DAPI (Vector Laboratories, # H-1200). Slides stored in the dark until image acquisition. At least three representative images of tumor sections were acquired and analyzed with Image J.

For in vitro immunofluorescent analyses, PAIII cells were seeded into 8-well chamber slides (LAB-TEK Cat #154534) at 2 × 10^4^ and cultured overnight before treatment with either 50% MSC CM or DMEM 5% FBS or 100 nM etoposide for 5 h. Cells were then rinsed with PBS and fixed in 4% PFA at room temperature for 20 min. Fixed cells were then blocked for 30 min at room temperature in antibody diluting buffer (2% BSA, 0.1% Triton X-100 in PBS). Primary antibodies (Cleaved Caspase 3, Cell Signaling Technology, #9661S), 1:400 dilution in antibody diluting buffer; Rabbit IgG Isotype Control, Thermo Scientific #31235) were incubated at room temperature for 30 min. Cells were then washed 3× in PBS and incubated with secondary antibody (Alexa FluorTM Goat Anti-Rabbit 488, Invitrogen #A-11034, 1:1000 dilution in antibody diluting buffer) for 30 min at room temp in the dark. After washing 3× in PBS, culture chambers were removed and the slides mounted with Vectashield Antifade Mounting Medium with Dapi (Vector Laboratories, # H-1200). Mounted slides were stored in the dark at 4 °C until microscopic analysis. Three representative images were acquired at ×40 magnification and the ratio of cleaved caspase positive to negative cells calculated with ImageJ.

### Hematoxylin and Eosin and, TRAP staining

Formalin-fixed paraffin-embedded tissue sections (5 μm) were stained with hematoxylin and eosin for histological analyses. For TRAcP staining, slides were incubated in buffer (112 mM anhydrous sodium acetate, 49 mM dibasic dehydrate sodium tartrate, 0.28% glacial acetic acid) containing 1% naphthol-phosphate substrate (2% napthol AS-BI phosphate in 2-ethoxyethanol) for 1 h at 37 °C. Slides were then transferred to buffer containing 250 μl of 5% pararosaniline dye in 2 N HCl and 250 μl of 4% sodium nitrite at 37 °C and monitored for development of red stained osteoclasts. Slides were rinsed in H_2_O, counterstained with hematoxylin, and aqueously mounted. The number of bone-lining, multi-nucleated (>3 nuclei per cell), TRAcP positive osteoclasts was quantified from multiple sections.

### Immunoblotting and STAT activity assays

Cells were lysed with RIPA (150 mM NaCl, 1 mM EDTA, 1% Triton X-100, 1% sodium deoxycholate, 0.1% SDS, 20 mM Tris, pH 8). Protein concentration was determined by BCA (Pierce, Waltham, MA, USA; #23225). Blots were blocked in 5% BSA for 1 h followed by primary antibody. All primary antibodies were purchased from Cell Signaling Technology (Cleaved Caspase 3 #9661S, pSTAT1 Tyr701 #7649S, pSTAT1 Ser727 #9177, pSTAT3 Tyr705 (D3A7) #9145, STAT3 (D3Z26) #12640S, STAT1 #9172S pJAK2 Y1007/1008 # 3776S), Santa Cruz (IL-10Rβ, Cat# sc271969) or Abcam (IL-28Rα, Cat# ab83865). Primary antibodies were diluted 1:1000 in blocking solution +0.1% Tween-20, and were incubated overnight at 4 °C. Beta Actin (Santa Cruz sc-1615 or Cell Signaling #3700) was used as a loading control. Blots were washed, then incubated with HRP-conjugated anti-species secondary (Cell Signaling Technology, Rabbit #7074/Mouse #7076, 1:1000) and developed by enhanced chemiluminescence followed by exposure to light-sensitive film or imaging by LI-COR Odyssey Fc. MSC CM was analyzed using Ray Biotech Mouse Cytokine Array C-2000 (AAM-CYT-2000) per manufacturer’s instructions. STAT1 and 3 DNA binding activity assays were performed per manufacturer’s lysis buffers (Active Motif, Cat #40010) and instructions (Active Motif, Cat #42296). All full scan blots are presented in supplied Source data files uploaded to *Nature Communications*.

### PCR gene expression

RNA was extracted with TRIzol (Invitrogen #15596) from F0 and F2 PaIII and F0 and F2 DU145 after treatment with MSC CM for 0, 1, 5, 10, and 60 min. Reverse transcription was performed using a High Capacity cDNA Reverse Transcription Kit (Applied Biosystems #4368813). PCR reactions using target specific primers (Supplemental Table S1) were set up using HotStarTaq Master Mix (Qiagen, #203445). PCR cycle conditions were 95 °C for 15 min, followed by 35 cycles (95 °C for 30 s, 57 °C for IL-28Rα and IL10Rβ or 55 °C for IL-28 and 18S; 30 s, 72 °C for 45 s.

### shRNA and siRNA of IL-28Rα and STAT3

Lentivirus expressing human or rat IL-28Rα shRNA (Origene, human; HT144125A-D, Rat; HT144126A-D, scrambled control; TR30033) were generated using standard procedures. PAIII and DU145 cells were seeded into 6 well plates (1 × 10^6^/well) and transduced with retrovirus in 4 μg/ml polybrene. After 24 h the media was replaced with 2 mls of 10% serum containing DMEM without antibiotics for 48 h to allow for recovery. Stable clones were selected via treatment with media containing blasticidin (8 µg/ml). Prior to analysis, IL-28Rα levels were analyzed via immunoblot as described. For STAT3 siRNA, 1 × 10^6^ cells from each cell line were seeded into individual 6 well plates and incubated overnight. Cells were transfected using lipofectomine RNAiMAX (Thermo Fisher Scientific # 13778500) with either rat (Origene; SR501698A-C), human (SR321907A-C) or control (SR2004) siRNAs according to manufacturer’s instructions. At 48 h post transfection, cells were trypsinized and seeded (3 × 10^3^) in triplicate in 96 well plates for growth analysis at 24, 48, and 72 h. Remaining cells were lysed and assessed by immunoblot as described for total STAT3.

### Statistical analysis

To determine statistical significance among groups, all sample sizes were greater than or equal to n of 3 and all measurements were taken from distinct samples. T-test or analysis of variance (ANOVA) followed by Tukey’s multiple comparison test was performed. A *p*-value <0.05 was considered as statistically significant. Data are presented as standard error from the mean (S.E.M). All statistical analyses were performed with Graph Pad Prism 6.0 (GraphPad Inc. LaJolla, CA). Further analysis of the data were performed by the Moffitt biostatistics and bioinformatics shared resource.

## Supplementary information

Supplementary Information

## Data Availability

The RNA QuantSeq data have been deposited in the NCBI Gene Expression Omnibus (GEO) database under the accession code GSE 163374 and are available at https://www.ncbi.nlm.nih.gov/geo/query/acc.cgi?acc=GSE163374. All the data supporting the findings of this study are available within the article and its Supplementary information files and from the corresponding author upon reasonable request. A reporting summary for this article is available as a Supplementary Information file. Source data are provided with this paper.

## Source data

Source Data
