# Supplementary material for: Mesenchymal stem cell-derived interleukin-28 drives the selection of apoptosis resistant bone metastatic prostate cancer
Source: Nat Commun. 2021 Feb 1;12:723. doi: 10.1038/s41467-021-20962-6 (PMC7851397; doi:10.1038/s41467-021-20962-6)
Supplement: Supplementary file 1 — Supplementary Information [file 41467_2021_20962_MOESM1_ESM.pdf]

Supplementary Information

Mesenchymal Stem Cell-Derived Interleukin-28 Drives the Selection of Apoptosis Resistant Bone Metastatic Prostate Cancer

Jeremy J. McGuire[1,2], Jeremy M. Frieling[2], Chen Hao Lo[1,2], Tao Li[2] Ayaz Muhammad[3], Harshani Lawrence[3], Nicholas Lawrence[3], Leah M. Cook[4], and Conor C. Lynch[2]*

[1]Cancer Biology Ph.D. Program, University of South Florida, Tampa, FL, USA, [2]Tumor Biology Department, [3]Department of Drug Discovery, H. Lee Moffitt Cancer Center and Research Institute, Tampa, FL, USA and [4]Department of Pathology and Microbiology, University of Nebraska Medical Center, Omaha, NE.

*Corresponding Author: Conor C. Lynch, Department of Tumor Biology, H. Lee Moffitt Cancer Center and Research Institute,12902, Magnolia Drive, Tampa, FL 33612. Phone 813-745-8094: Fax: 813-745-3829; E-mail: conor.lynch@moffitt.org

SUPPLEMENTARY MATERIALS AND METHODS

MSC characterization by FACS analysis

The Mouse Mesenchymal Stem Cell Multi-Color Flow Kit (R&D Cat # FMC003) was used according to manufacturer's protocol. Isolated MSCs were assessed by flow cytometry for CD29 and SCA-1 positivity and CD45 negativity. For FACs analysis 1X106 MSCs were suspended in flow cytometry staining buffer. For each marker 90µl of the cell suspension were transferred to 5ml flow tubes and incubated for 30 minutes with each antibody or isotype control. Cells were then washed twice in 2 ml of flow cytometry staining buffer and resuspended in 200µl of the same buffer for analysis.

Differentiation assays

Osteogenic differentiation and staining: MSCs grown to confluency before treatment with 20X murine osteogenic supplement (R&D Cat# CCM009) added to normal growth medium and changed every 2-3 days for 21 days. Negative controls were grown in normal growth medium. Cells were then stained with alizarin red (Fisher scientific Cat # 130-22-3). Cells were washed gently three times in PBS and fixed for 15 minutes using 10% buffered formalin. Fixed cells were washed two times with ddH20 and then stained in the dark for 45 minutes in 2% alizarin red solution. (Note pH is critical for 2% alizarin red solution pH 4.2-4.3 adjusted using 10% ammonium hydroxide). Cells were washed gently 4 times with ddH20 and air dried before images were taken. For adipogenic differentiation and staining: MSCs were grown to confluency and then treated with 100X Adipogenic supplement (R&D Cat# CCM011) in normal growth medium or negative control in normal growth medium changed every 2-3 days for 21 days. Cells were stained with Oil Red O. (Sigma Cat# 01391). Cells were washed with PBS and then stained using 1 part Oil Red O 1 part water for 15 minutes. Cells washed three times with ddH2O and imaged for red oil droplets. For chondrogenic differentiation and staining: MSCs were trypsinized and counted 1.5X105 cells were spun down at 200g for 5 min in six 15 ml conical tubes. The media was then replaced with media containing 100X chondrogenic supplement (R&D Cat# CCM006) or just base media in triplicate. The caps of the tubes were loosened and incubated at 37°C. After two days the cells formed small spheres and the media was replaced every 2-3 days for 28 days. The pellets were then washed in PBS and embedded in paraffin for histology. Consecutive slides were H&E stained and the second slide stained with alcian blue (Sigma Cat # B8438). For alcian blue staining slides were dewaxed in xylenes rehydrated to water and stained for 30 min in alcian blue solution. Slides were then washed in running tap water for two minutes followed by a rinse in H2O. Slides were

then dehydrated to 100% ethanol and cleared in xylenes before mounting cover slips with permount and imaging.

Analysis of publically available datasets

IL-28 (1), IL-28Rα (2) and IL-10Rβ (3) expression levels in control (prostate gland tissue) or prostate adenocarcinoma were performed using the Oncomine platform (www.oncomine.org, (4)).

FasL studies

For PAIII treatment 6X10^4 cells were seeded in 48 well plates in triplicate and treated with murine rFasL (R&D Cat # 6128-SA/CF) at varying concentrations. Anti-HA antibody (2.5ug/ml) was used to trimerize and activate the FasL (R&D Cat #MAB060) incubated with or without rFasL at 4C for 1 hour while mixing. Subsequent to 48 hours incubation, cells were lysed and luminescence was quantified using the Promega luciferase assay. For cleaved caspase-3 immunofluorescence studies, PAIIIs (F0 or F2) were seeded into 8-well chamber slides (LAB-TEK #154534) at 2x104 and cultured overnight before treatment with anti-HA alone, 10ng/ml rFasL+HA or 100nM etoposide for 5 hours. Cells were then rinsed with PBS and fixed in 4% PFA at room temperature for 20 minutes. Fixed cells were then blocked for 30 minutes at room temperature in antibody diluting buffer (2% BSA, 0.1% Triton X-100 in PBS). Primary antibodies Cleaved Caspase 3,(Cell Signaling Technology #9661S), 1:400 dilution in antibody diluting buffer; Rabbit IgG Isotype Control, (Thermo Scientific #31235) were incubated at room temperature for 30 minutes. Aspirate and Cells were then washed 3x in PBS and incubated with secondary antibody (Alexa FluorTM Goat Anti- Rabbit 488,(Invitrogen #A-11034), 1:1000 dilution in antibody diluting buffer for 30 minutes at room temp in the dark. After washing 3x in PBS, culture chambers were removed and the slides mounted with Vectashield Antifade Mounting Medium with Dapi (Vector Laboratories, # H-1200). Ten representative images from each group were counted and the number of CC3+ cells were plotted as a percentage to the total number of DAPI stained nuclei.

QuantSeq analysis

Total RNA was extracted using biological replicates collected in triplicate from each, F0 and F2 PaIII and F0 and F2 DU145 cell lines using the RNeasy kit (Qiagen Cat# 74104) followed by DNase treatment using the Max Kit (Qiagen Cat # 15200). RNA concentrations were measured using the Qubit RNA BR assay and RNA integrity was assessed using the Agilent 4200 Tapestation. 500ng of RNA per sample was processed by the Moffitt Molecular Genomics Core to generate libraries for gene expression analysis using the Lexogen QuantSeq 3' mRNA-Seq

Library Prep Kit FWD for Illumina (Lexogen Cat #015) following the manufacturer's protocol. At least 20 million 101-base single-end sequencing reads per sample were generated on the Illumina NextSeq 500 sequencer. The Bluebee Genomics Platform provided with the QuantSeq kit from Lexogen was used for read for alignment, counting, and differential expression analysis.

Supplementary Figures

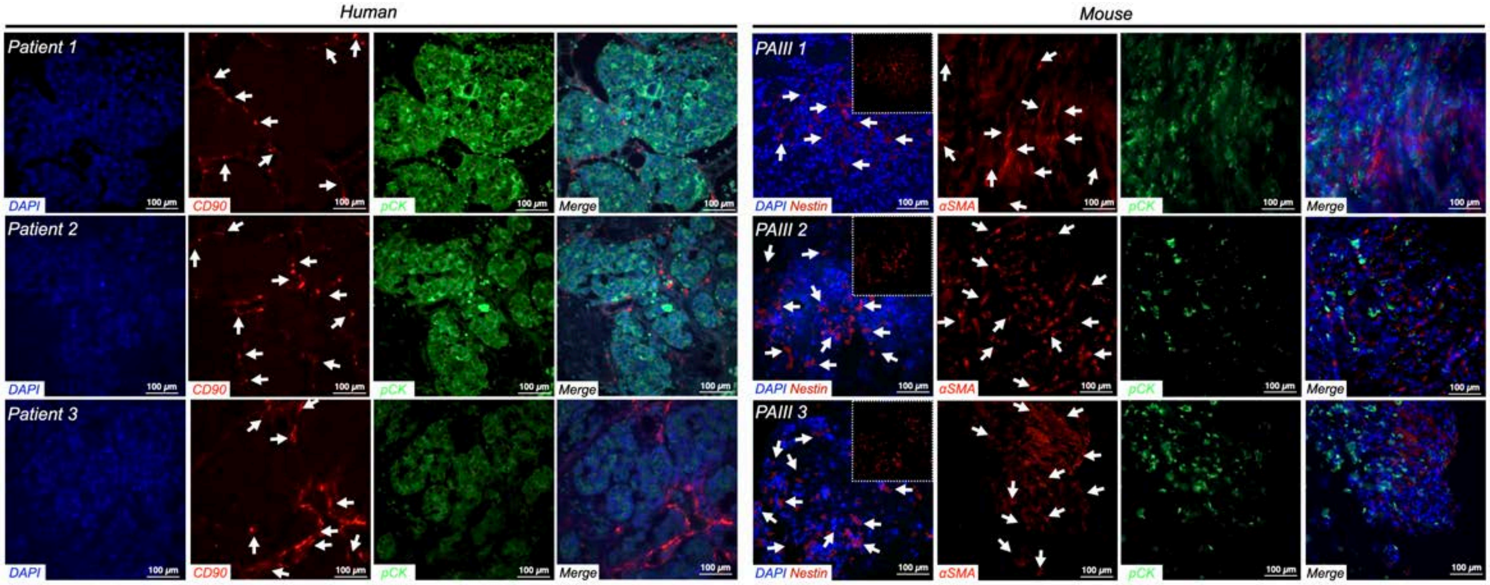

Supplementary Figure 1. MSC infiltration in bone metastatic prostate cancer. Representative images of CD90 staining (red; arrows) in human samples of bone metastatic prostate (n=10; three representative patients are shown) or nestin staining (red; arrows) in tissue sections derived from mouse tibias bearing PAIII bone metastases (three representative mice are shown). For human and mouse specimens, pan-cytokeratin (pCK; green) was used to localize prostate cancer cells while DAPI (blue) was used as a nuclear stain. Dashed box in merge represents area of magnification.

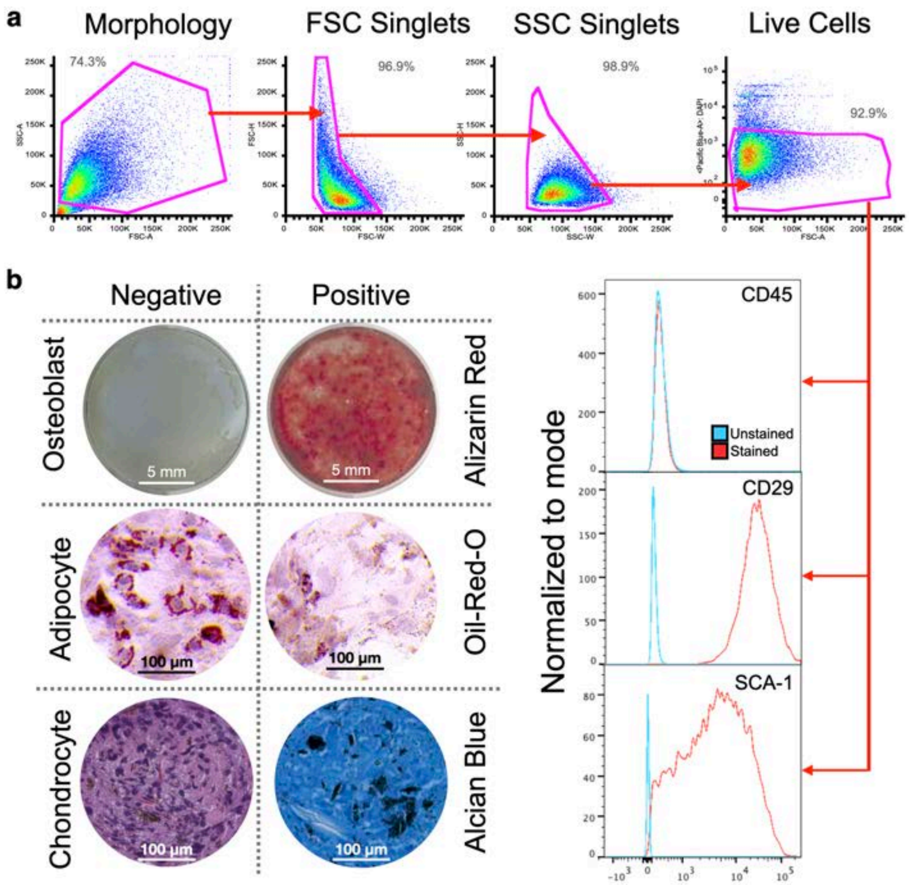

Supplementary Figure 2. Characterization of mouse isolated MSCs. Flow cytometry gating strategy to identify MSCs. MSCs were characterized using FLOW cytometry for CD29 and SCA1 positivity and CD45 negativity. The MSCs were then differentiated into osteoblasts stained with alizarin red, adipocytes stained with oil red o and chondrocytes stained with alcian blue all differentiation assays were repeated in triplicate with similar results.

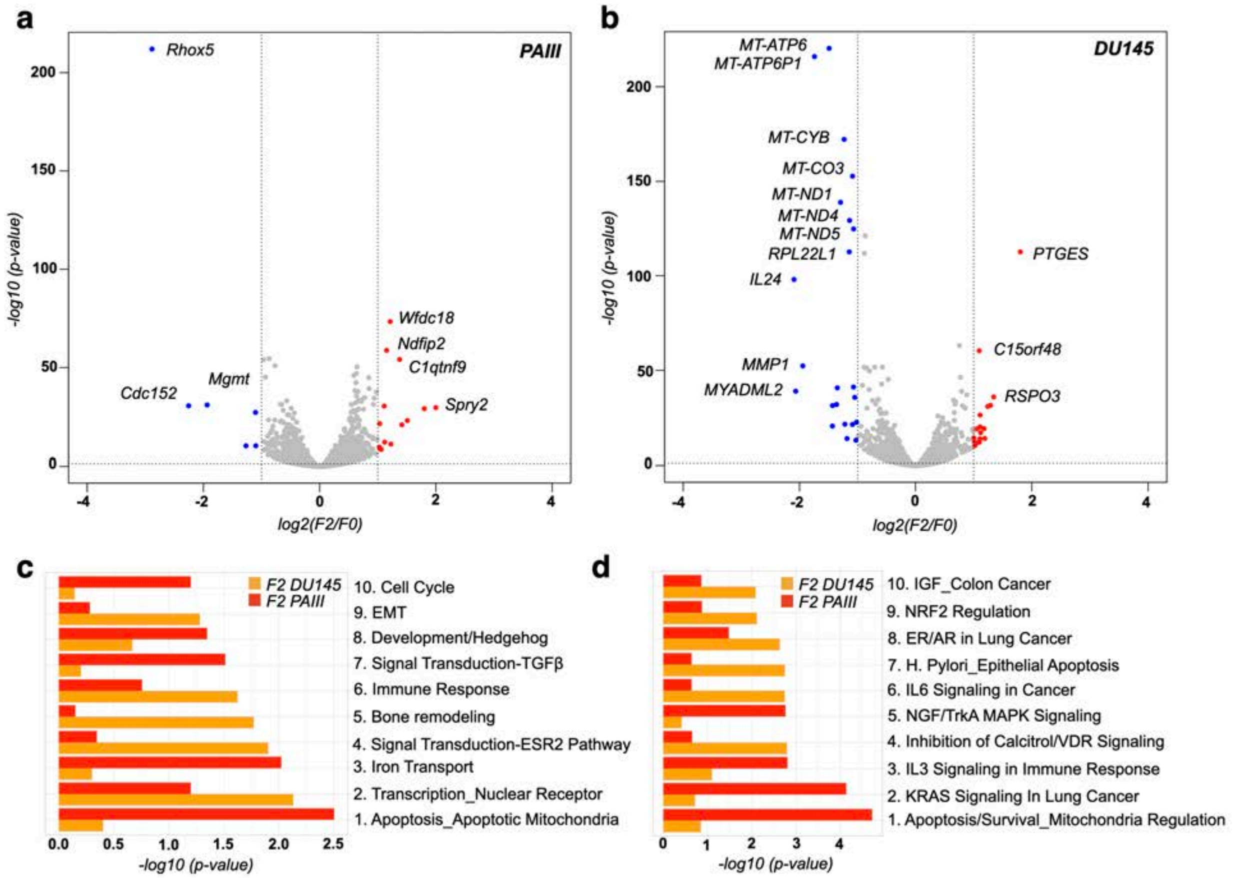

Supplementary Figure 3. RNA Quant profile of MSC educated PAIII and DU145 cell lines. a, b, Volcano plot showing log2 fold changes RNA profile between in the F2 PAIII (a) and DU145 (b) cells compared to their respective F0 controls. **c, d,** Network (c) and pathway (d) analyses of the RNA profiles in the F2 PAIII and DU145 compared to their respective F0 controls.

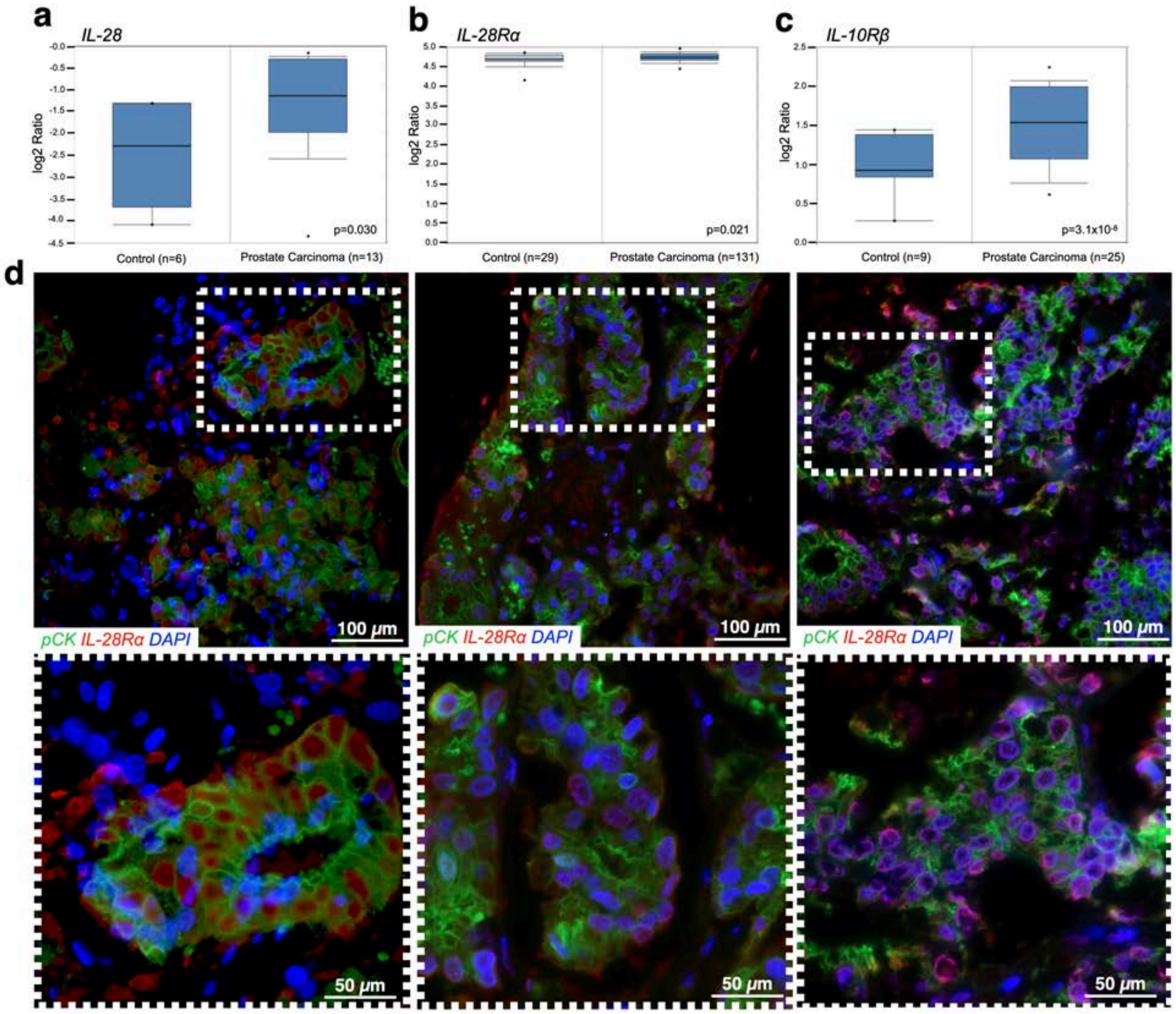

Supplementary Figure 4. IL-28Rα expression in human prostate cancer. **a-c,** Public database analysis of IL-28 (a), IL-28Rα (b) and IL10Rβ (c) in human prostate gland (control) and primary prostate cancer tissue. Box and whisker plots shown mean with min and max. **d,** Representative images of IL-28Rα staining (red) in human samples of bone metastatic prostate (n=10; three representative images are shown). Pan-cytokeratin (pCK; green) was used to localize prostate cancer cells while DAPI (blue) was used as a nuclear stain. Dashed box in merge represents area of magnification.

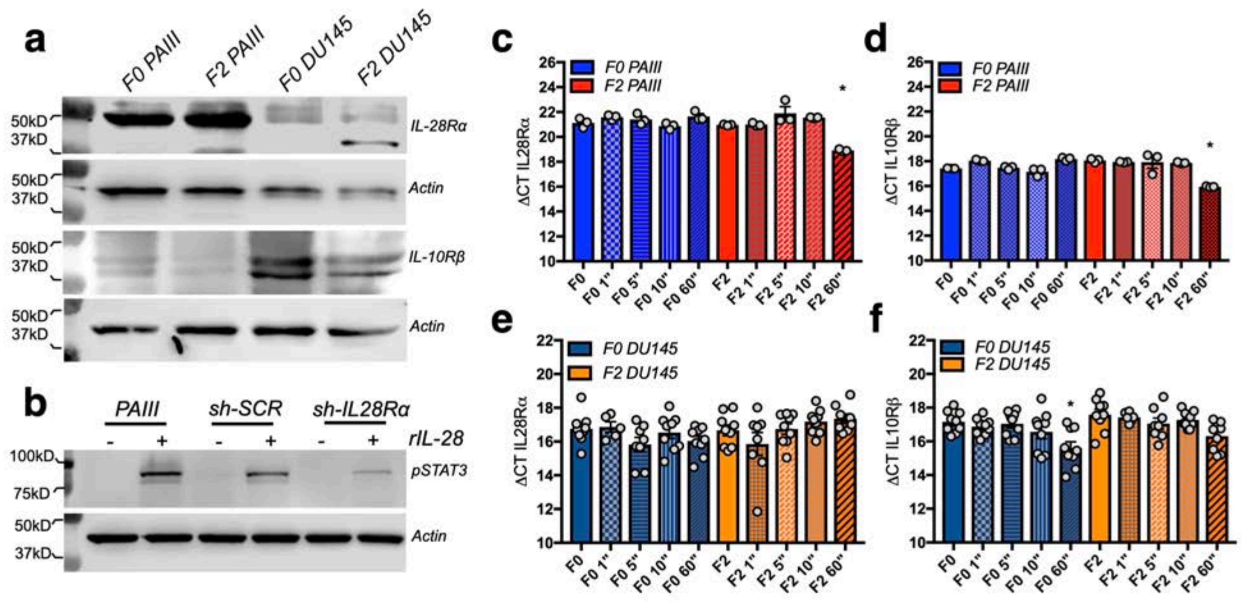

Supplementary Figure 5. IL-28Rα and IL-10Rβ expression in F0 and F2 PAIII and DU145 cell lines. **a,** IL-28Rα and IL-10Rβ expression in F0 and F2 PAIII and DU145 cell lines. **b,** STAT3 phosphorylation in PAIII parental (PAIII), scrambled control (sh-SCR) and IL-28Rα silenced (sh-IL28Rα) cell lines in response to treatment with recombinant IL-28 (rIL-28; 25ng/ml) for 15 minutes. Actin was used a loading control. Molecular weights are shown in kDa. **c-f**, Realtime qPCR analysis of IL28Rα and IL10Rβ in F0 and F2 PAIII (c, d) and DU145 (e, f) cells lines in response to MSC CM at 1, 5, 10 and 60 minutes. **a.b** repeated two times with similar results. **c.d.** n=3 biologically independent samples **e.f** n=3 biologically independent samples run in triplicate. Statistical analysis one-way ANOVA with multiple comparisons 95% CI. Asterisk denotes statistical significance (*p≤0.05).

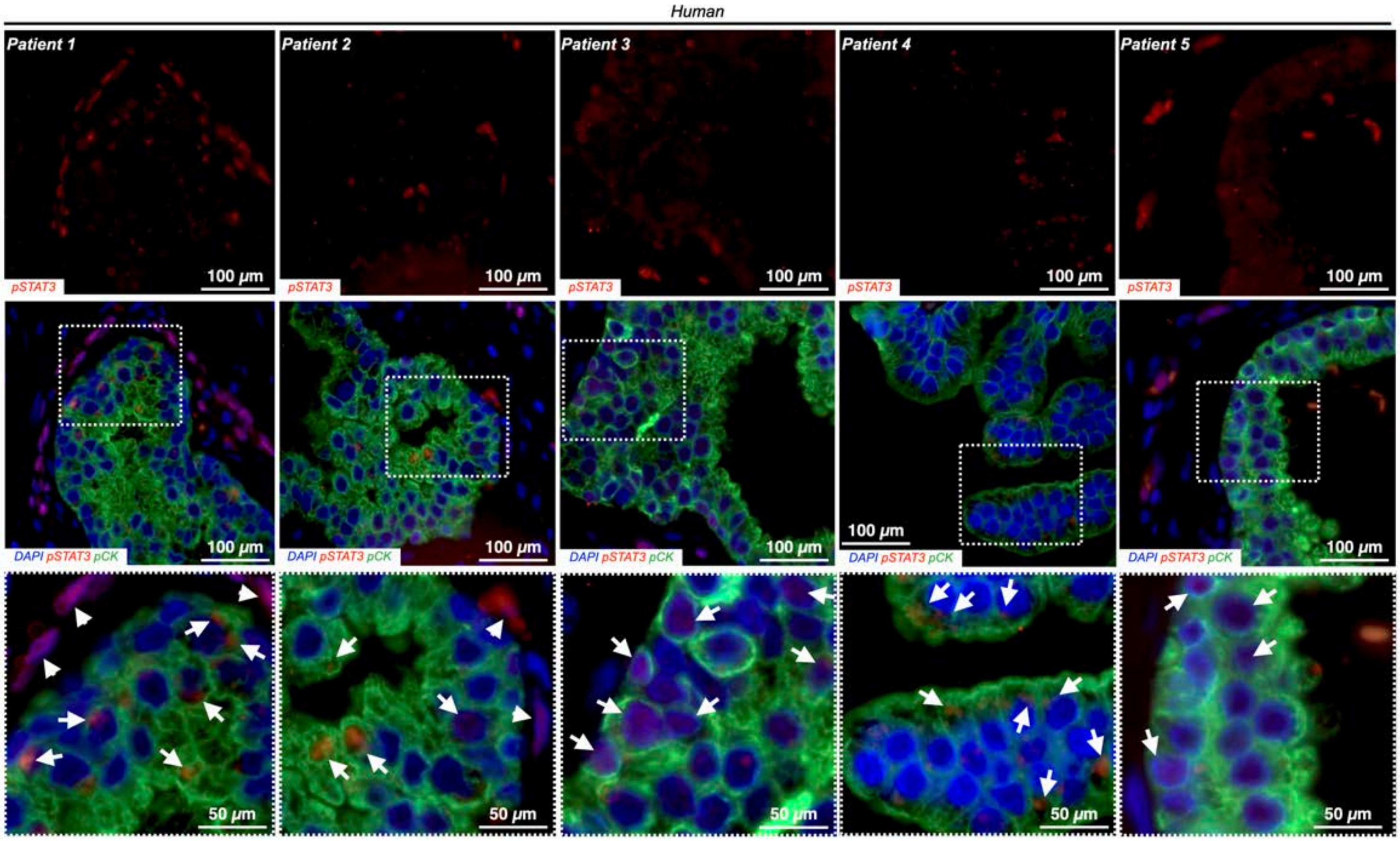

Supplementary Figure 6. pSTAT3 localization in human bone metastatic prostate cancer specimens. Representative images of pSTAT3 staining (red) in human samples of bone metastatic prostate cancer (n=10; five representative patients are shown). Pan-cytokeratin (pCK; green) was used to localize prostate cancer cells while DAPI (blue) was used as a nuclear stain. Dashed box in merge represents area of magnification. Arrows used to identify pSTAT3 staining in prostate cancer cells while arrow heads identify positive staining in the supporting bone stromal cells.

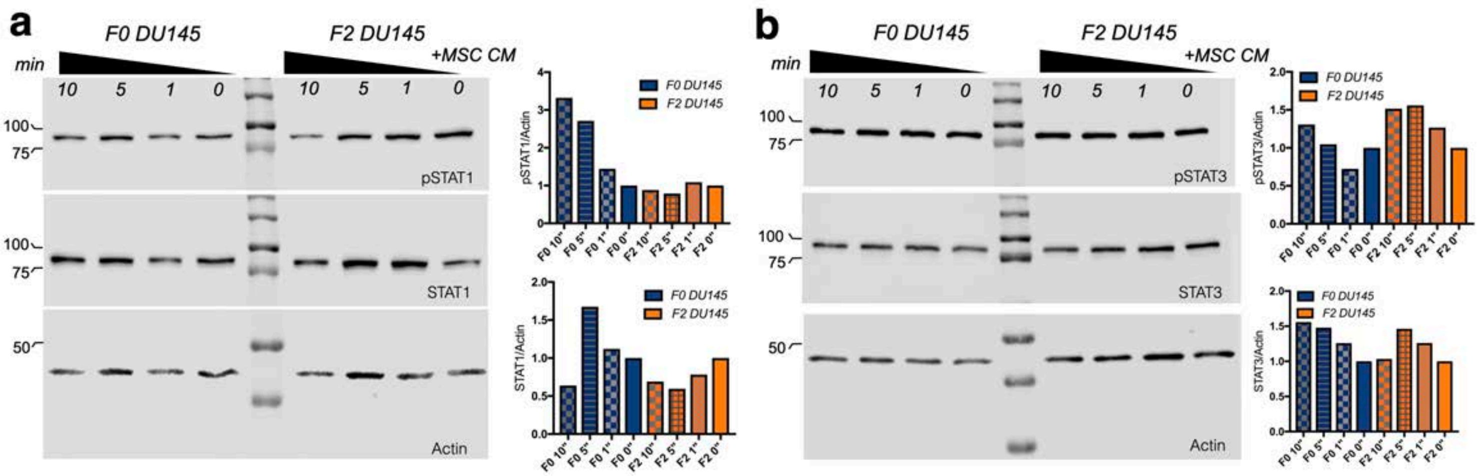

Supplementary Figure 7. Elevated STAT3 signaling in MSC selected prostate cancer cell lines. a, b, pSTAT1 (a) and pSTAT3 (b) levels at baseline and in response to MSC CM (50%) over a 10 minute (min) period in DU145 parental (F0) and MSC selected (F2) cell lines. Molecular weights are shown in kDa. Actin was used as a loading control. For densitometry, pSTAT and STAT levels were each normalized to their respective actin controls and time responses for F0 and F2 are compared to their respective 0' minute controls that were set at a normalized value of 1. All blots were run in triplicate with similar results.

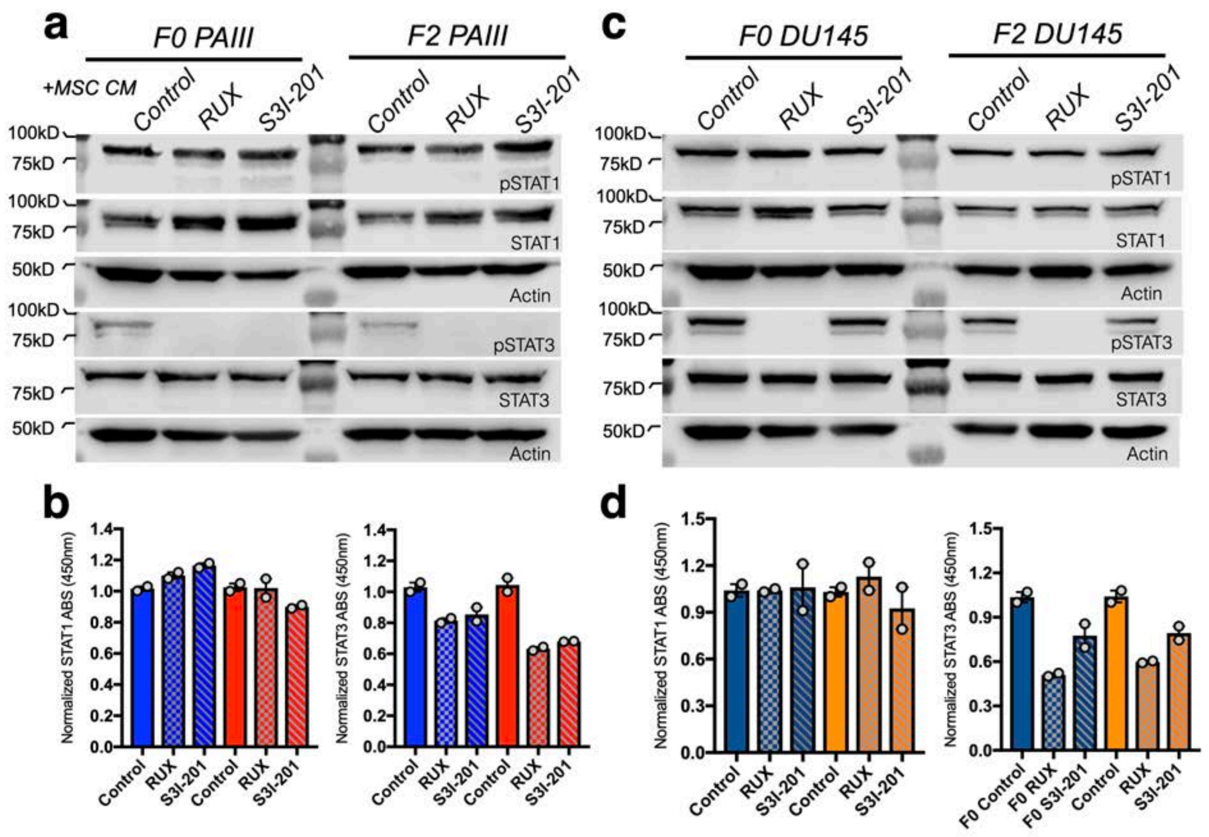

Supplementary Figure 8. STAT3 phosphorylation status in response to ruxolitinib and S3I-201. a, b STAT3 and JAK2 phosphorylation was assessed in response to ruxolitinib (Rux; 100nM) and S3I-201 (100μM) treatment for 6 hours in F0 and F2 PAIII (a) and DU145 (b) cell lines in the presence of MSC CM. Actin was used as a positive loading control. Molecular weights are shown in kDa. **c, d,** STAT1 and STAT3 DNA binding activity in the PAIII (c) and DU145 (d) F0 and F2 cell lines was measured in response to MSC CM for 30 minutes. Results obtained via absorbance (ABS@450nm) were normalized to respective controls. **a,c,** Blots were run in triplicate with similar results.

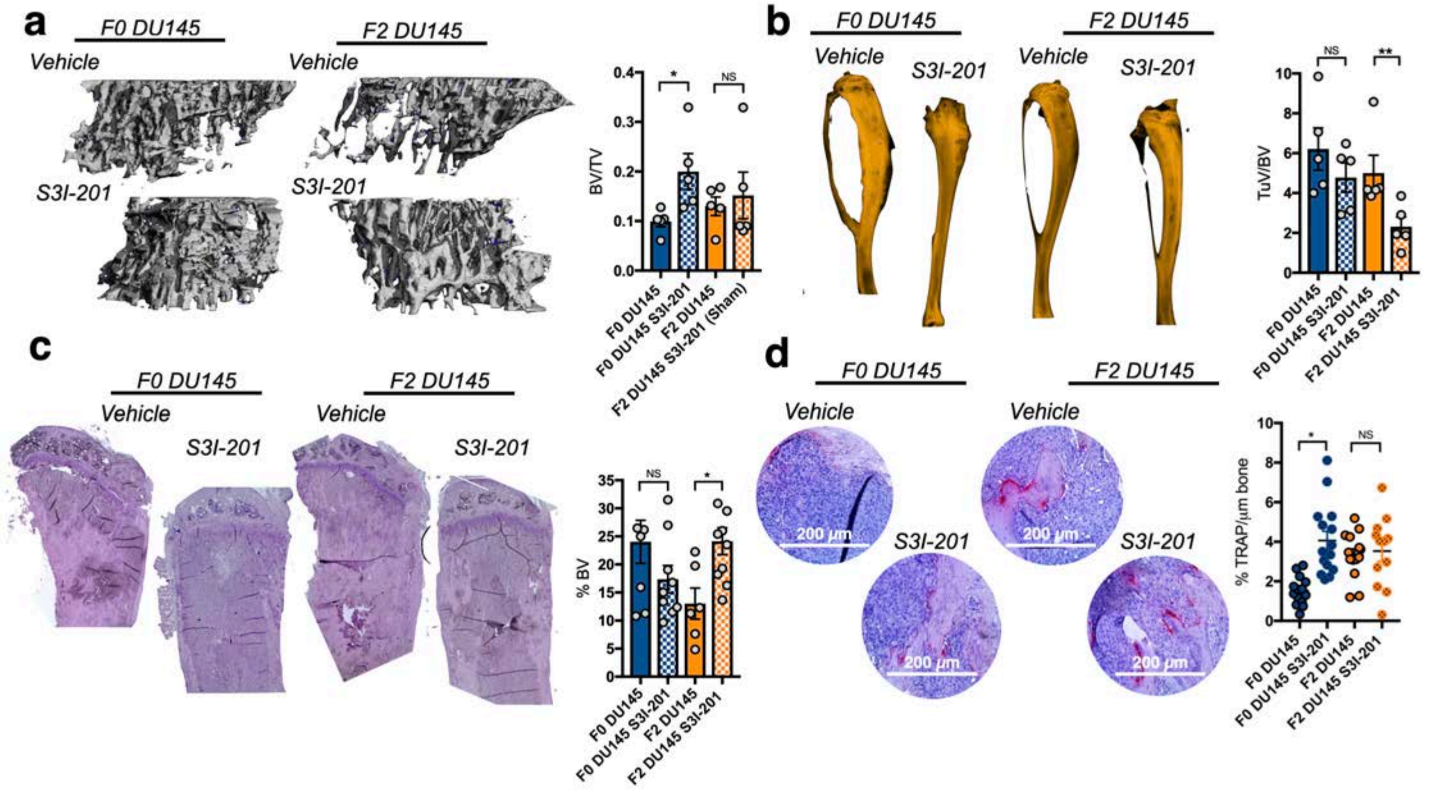

Supplementary Figure 9. STAT3 impacts prostate cancer associated bone disease in the F0 and F2 DU145 model. **a,** μCT scan analysis of cancer-induced bone destruction. Representative μCT images of the trabecular bone are shown for the vehicle alone and S3I-201 F0 and F2 groups. The trabecular bone volume was calculated as a ratio to total volume analyzed (BV/TV). n≥4 bones from each group **b,** Faxitron X-ray analysis of cancer-induced osteolytic lesions. Representative images of the X-rays are shown for each group. The size of the tumor volume as measured by the area of the lytic lesion (TuV) was calculated as a ratio to total volume analyzed (TuV/TV). n=5 bones from each group **c,** Trabecular bone volume (BV) was measured via histomorphometry on non-sequential hematoxylin and eosin (H&E) multiple sections derived from each group and calculated as a percentage of total volume. Representative gross H&E images are shown for the vehicle and S3I-201 F0 and F2 groups. n≥3 bones from each group **d,** The number of osteoclasts (TRAcP positive; red, multi-nucleated; arrows) per μm of bone was calculated in non-sequential sections derived from each group. Representative gross TRAcP images are shown for the vehicle and S3I-201 F0 and F2 groups. n=3 representative images from n=3 different bones. **a-d** Error bars are mean +/- SEM statistical analysis one-way ANOVA with multiple comparisons 95% CI. Asterisks denotes statistical significance (*p≤0.05, **p≤0.01, ***p≤0.001, ****p≤0.0001) while NS denotes not significant.

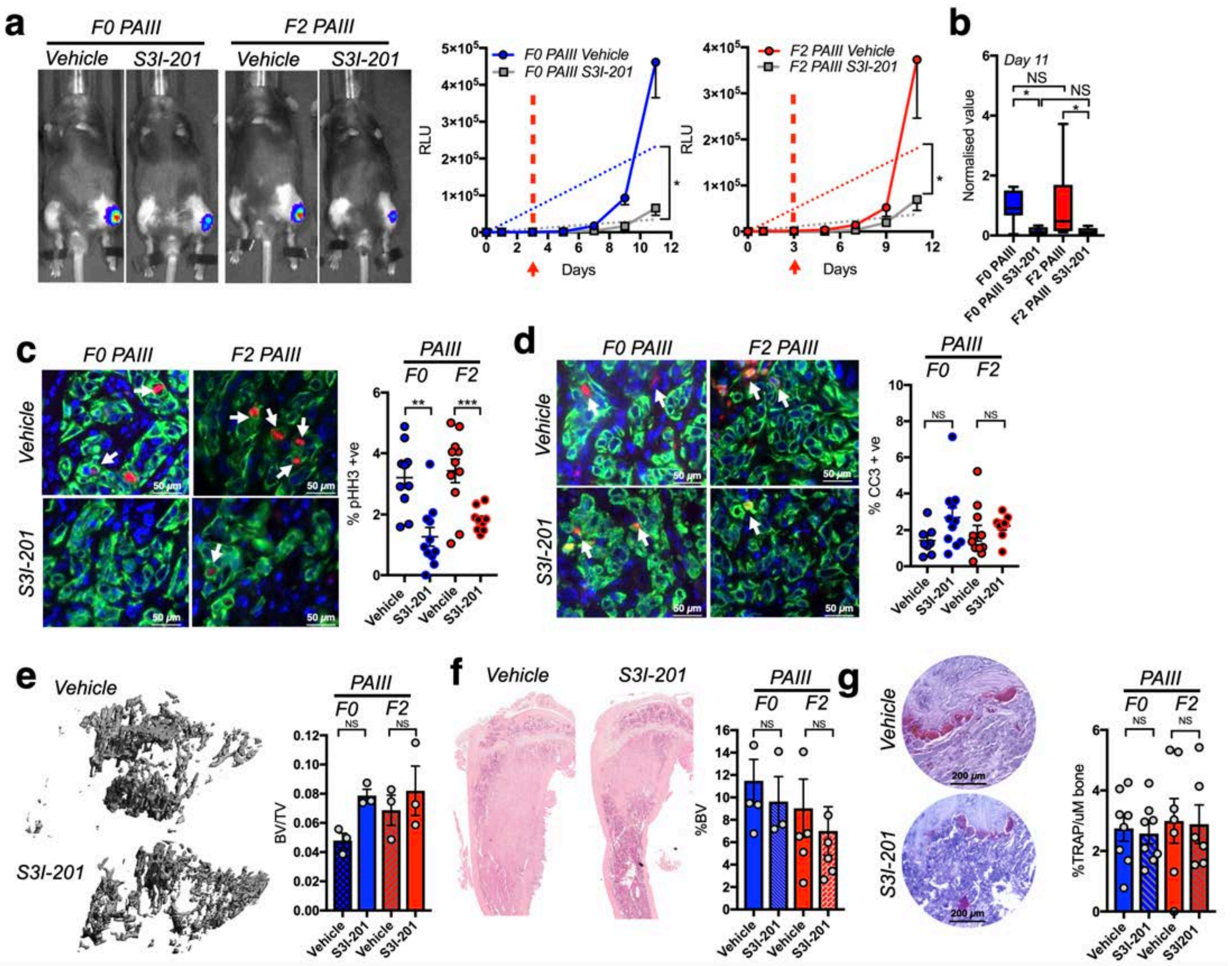

Supplementary Figure 10. STAT3 inhibition impairs the growth of MSC-selected prostate cancer *in vitro* and *in vivo*. a, F0 and F2 PAIII growth over time in the presence or absence of STAT3 inhibitor, S3I-201 (n≥8/group). Representative images of bioluminescence in each group are shown at day 11 time point. Arrow and dashed line represent time of treatment initiation. Graphs illustrate collected RLUs over time for each group. Error bars are mean +/- SEM with dotted lines representing linear regression used for statistical analysis. **b,** S3I-201 effect on F0 and F2 PAIII at day 11 normalized to respective controls. Box and whisker plots are mean +/- SEM. **c, d** *Ex vivo* analyses from study endpoint of proliferative and apoptotic indices using phospohistone H3 (pHH3; red arrows; b) and cleaved caspase 3 (CC3; red, arrows, c) respectively. Pan-cytokeratin (green) was used to identify prostate cancer cells. n=3 representative images from n=3 different bones. **e,** μCT scan analysis of cancer-induced bone destruction. Representative μCT images of the trabecular bone are shown for the vehicle alone

and S3I-201 F0 groups. The trabecular bone volume was calculated as a ratio to total volume analyzed (BV/TV). n=3 different bones. **f,** Trabecular bone volume (BV) was measured via histomorphometry on non-sequential hematoxylin and eosin (H&E) multiple sections derived from each group and calculated as a percentage of total volume. Representative gross H&E images are shown for the vehicle and S3I-201 F0 groups. n≥3 different bones. **g,** The number of osteoclasts (TRAcP positive; red, multi-nucleated; arrows) per µm of bone was calculated in non-sequential sections derived from each group. Representative gross TRAcP images are shown for the vehicle and S3I-201 F0 groups. n=1-3 representative images from n=3 different bones. **b-g,** Stat nistical analysis one-way ANOVA with multiple comparisons. 95% CI. Asterisks denotes statistical significance (*p≤0.05, **p≤0.01, ***p≤0.001, ****p≤0.0001) while NS denotes not significant.

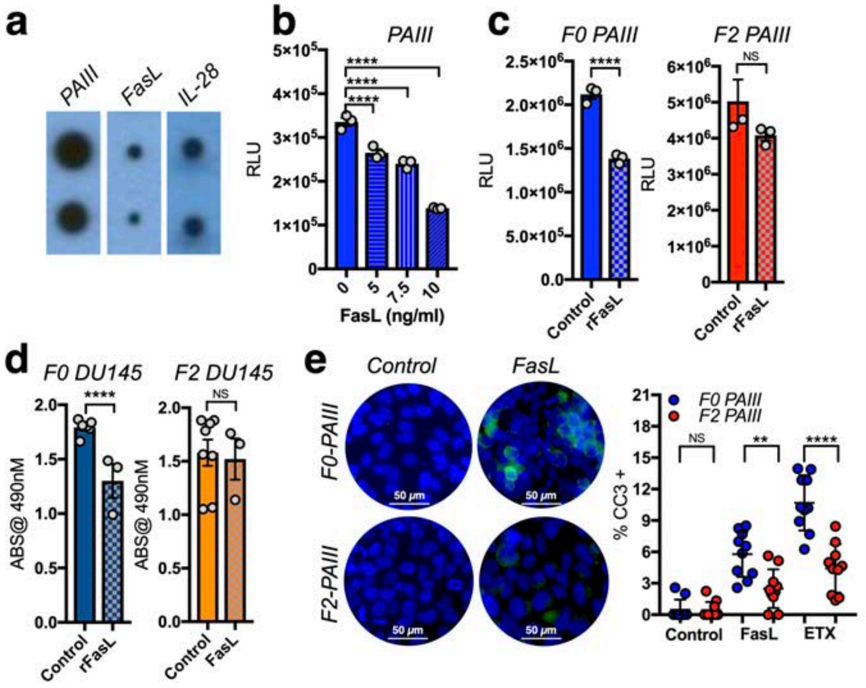

Supplementary Figure 11. MSC derived FasL promotes PCa apoptosis. a, Cytokine Array of MSC CM. Positive control and levels of FasL and IL-28 are illustrated. Cytokine Array of MSC CM. Black box indicates positive control, red box indicates IL-28. **b,** PAIII response to increasing concentrations of FasL for 48 hours. Anti-HA alone was used as a control. **c, d,** F0 and F2 PAIII and DU145 cell lines treated in the presence or absence of recombinant FasL (10ng/mL) for 48 hours. **e,** Immunofluorescence (IF) analysis of cleaved caspase-3 positivity (green) in F0 (d) and F2 (d) PAIII cell lines treated for 6 hours with rFasL. Graphs illustrate the number of cleaved caspase-3 positive cells as a ratio of total cell number (nuclear DAPI-blue) per multiple fields of view. Etoposide (ETX; 50μM) was used as a positive control. n=1-3 fields of view from n=3 different bones. **b-d,** n≥3 biologically independent samples **b-e,** Error bars are mean +/- SEM one-way ANOVA with multiple comparisons 95% CI used for statistical analysis. Asterisks denotes statistical significance (**p≤0.01, ****p≤0.0001) while NS denotes not significant.

Primer	Forward	Reverse
Mouse IL-28	*5′-GTTCAAGTCTCTGTCCCCAAA -3′*	*5′-GTGGAACTGCACCTCATGT-3′*
Rat IL-28Rα	*5′-CCTGTTCCTGATGCAAAGCG-3′*	*5′-AAGTAGGTCACATTCGGGGG-3′*
Rat IL-10Rβ	*5′-GAACGGGAGAGTGGAGCAAA -3′*	*5′-ATGCTGAAGCAGCCCAGTAG-3′*
Human IL-10RB	*5′-GGAATGGAGTGAGCCTGTCTGT-3′*	*5′-AAACGCACCACAGCAAGGCGAA-3′*
Human IL-28Rα	*5′-CAGCAAGTTCTCTAAGCCCACC-3′*	*5′-GTCATTCACGGACTCTGGTCTG-3′*
18S	*5′-GTAACCCGTTGAACCCCATT-3′*	*5′-CCATCCAATCGGTAGTAGCG-3′*

Supplemental Table 1. Table of the primers used to determine gene expression via RT-PCR.

SUPPLEMENTARY REFERENCES

1. Varambally S, Yu J, Laxman B, Rhodes DR, Mehra R, Tomlins SA, Shah RB, Chandran U, Monzon FA, Becich MJ, Wei JT, Pienta KJ, Ghosh D, Rubin MA, Chinnaiyan AM. Integrative genomic and proteomic analysis of prostate cancer reveals signatures of metastatic progression. Cancer Cell. 2005;8(5):393-406. doi: 10.1016/j.ccr.2005.10.001. PubMed PMID: 16286247.

2. Taylor BS, Schultz N, Hieronymus H, Gopalan A, Xiao Y, Carver BS, Arora VK, Kaushik P, Cerami E, Reva B, Antipin Y, Mitsiades N, Landers T, Dolgalev I, Major JE, Wilson M, Socci ND, Lash AE, Heguy A, Eastham JA, Scher HI, Reuter VE, Scardino PT, Sander C, Sawyers CL, Gerald WL. Integrative genomic profiling of human prostate cancer. Cancer Cell. 2010;18(1):11-22. doi: 10.1016/j.ccr.2010.05.026. PubMed PMID: 20579941; PMCID: PMC3198787.

3. Welsh JB, Sapinoso LM, Su AI, Kern SG, Wang-Rodriguez J, Moskaluk CA, Frierson HF, Jr., Hampton GM. Analysis of gene expression identifies candidate markers and pharmacological targets in prostate cancer. Cancer Res. 2001;61(16):5974-8. PubMed PMID: 11507037.

4. Rhodes DR, Kalyana-Sundaram S, Mahavisno V, Varambally R, Yu J, Briggs BB, Barrette TR, Anstet MJ, Kincead-Beal C, Kulkarni P, Varambally S, Ghosh D, Chinnaiyan AM. Oncomine 3.0: genes, pathways, and networks in a collection of 18,000 cancer gene expression profiles. Neoplasia. 2007;9(2):166-80. PubMed PMID: 17356713; PMCID: PMC1813932.
